# Mitochondrial stress-induced H4K12 hyperacetylation dysregulates transcription in Parkinson’s disease

**DOI:** 10.3389/fncel.2024.1422362

**Published:** 2024-08-12

**Authors:** Minhong Huang, Huajun Jin, Vellareddy Anantharam, Arthi Kanthasamy, Anumantha G. Kanthasamy

**Affiliations:** ^1^Parkinson Disorders Research Laboratory, Iowa Center for Advanced Neurotoxicology, Department of Biomedical Sciences, Iowa State University, Ames, IA, United States; ^2^Isakson Center for Neurological Disease Research, Department of Physiology and Pharmacology, University of Georgia, Athens, GA, United States

**Keywords:** mitochondrial dysfunction, Parkinson patients, acetylation, biomarker, aging, epigenomics

## Abstract

Aberrant epigenetic modification has been implicated in the pathogenesis of Parkinson’s disease (PD), which is characterized by the irreversible loss of dopaminergic (DAergic) neurons. However, the mechanistic landscape of histone acetylation (ac) in PD has yet to be fully explored. Herein, we mapped the proteomic acetylation profiling changes at core histones H4 and thus identified H4K12ac as a key epigenomic mark in dopaminergic neuronal cells as well as *in* MitoPark animal model of PD. Notably, the significantly elevated H4K12ac deposition in post-mortem PD brains highlights its clinical relevance to human PD. Increased histone acetyltransferase (HAT) activity and decreased histone deacetylase 2 (HDAC2) and HDAC4 were found in experimental PD cell models, suggesting the HAT/HDAC imbalance associated with mitochondrial stress. Following our delineation of the proteasome dysfunction that possibly contributes to H4K12ac deposition, we characterized the altered transcriptional profile and disease-associated pathways in the MitoPark mouse model of PD. Our study uncovers the axis of mitochondrial impairment-H4K12ac deposition-altered transcription/disease pathways as a neuroepigenetic mechanism underlying PD pathogenesis. These findings provide mechanistic information for the development of potential pharmacoepigenomic translational strategies for PD.

## Introduction

Parkinson’s disease (PD) is a progressive neurodegenerative disorder with major etiological association with aging and environmental risk factors. Approximately 10 million patients worldwide suffer from PD’s long-term economic and psychological burden, with roughly 60,000 more diagnosed in the U.S. each year. Epidemiology reports demonstrate a dramatically increasing trend worldwide in PD prevalence (Liu et al., 2016; Savica et al., 2016). Despite accumulating evidence pointing out that PD is a major societal concern, some current healthcare systems are in capable of meeting the projected needs of PD patients (Chen, 2016).

PD symptoms include both motor (e.g., bradykinesia, resting tremor, rigidity) and non-motor dysfunction (e.g., hyposmia, sleep disorders, depression, constipation, early satiety, and excessive sweating) (Sveinbjornsdottir, 2016; Schapira et al., 2017). So far, PD has no cure, and the current treatment is solely to abate symptoms. It is important to advance our understanding of pathological events in irreversible brain deterioration and to untangle the complex gene-environment interactions contributing to neurodegeneration. Epigenetic regulation has been implicated to play an essential role in modulating the risk and progression of PD (Song et al., 2010, 2011; Huang et al., 2022). The dynamic nature of epigenetic marks is exemplified by their ability to integrate the memory of chronic exposure to environmental stimuli into the genome. Clinically, epigenetic marks represent potentially tractable targets for PD treatment (Gräff and Tsai, 2013; Hegarty et al., 2016; Hwang et al., 2017; Jakubowski and Labrie, 2017; Berdasco and Esteller, 2019), especially for expanding the opportunities for precise personalized PD treatments tailored to individualized epigenomic profiles. Employing such pharmacoepigenomics could improve the predictability of epigenetic-based variation in individual drug response (Majchrzak-Celińska and Baer-Dubowska, 2017; Peedicayil, 2019). Recently, several cohorts of PD patients have demonstrated detectable alterations in epigenomic modifications from more accessible tissue, e.g., blood and saliva (Henderson-Smith et al., 2019; Nabais et al., 2021), further supporting the potential feasibility of pharmacoepigenomic approaches.

Few histone (H) acetylation (ac) analyse with a limited number of PD patients have been reported (Gebremedhin and Rademacher, 2016; Park et al., 2016; Toker et al., 2019) showing changes in specific acetylation sites from either midbrain tissues, primary motor cortex, or prefrontal cortex. A recent publication collected peripheral blood samples from 1,474 PD patients and 1,456 healthy controls for investigating the pathological role of dysfunctional mitochondrial metabolism. The study showed that mitochondrial aconitase 2 (ACO2) deficiency in TCA cycle increases vulnerability to PD by dysregulating histone aceytlation-mediated transcription of autophagy genes (Zhu et al., 2023). Mounting evidence highlights a pathological imbalance between histone acetylation-mediated histone acetyl transferease (HAT) and deacetylation by histone deacetylase (HDAC) in PD models, through which gene expression is dysregulated and downstream function is subsequently altered in DAergic neurons (Song et al., 2010; Labbé et al., 2016). Our recent study characterizing the important role of H3K27ac in the pathogenesis of PD links H3K27ac to mitochondrial dysfunction and subsequent changes in promoter modification and gene expression (Huang et al., 2021). However, the exact acetylation patterns of core histones H4 and their molecular mechanism in PD have yet to be defined. Therefore, the current study focuses on the PD-associated acetylation of core histone H4 to explore its potential epigenetic mark signature using cell and animal models and then correlate with human substantia nigra (SN) tissue, the hallmark brain region of PD neuropathology.

As such, we first used a genome-wide proteomic acetyl-site screening method to identify the dominant acetylation site on histone H4 that is sensitive to mitochondrial dysfunction induced by rotenone in DAergic neuronal N27 cells. Next, to examine how the identified H4 acetylation site responded in other experimental PD cell models, the MPTP analogue paraquat and another mitochondrial complex I inhibitor, pyridaben, were administered to N27 cells (Charli et al., 2016). Additionally, an N27 cell model stably expressing a mitochondrial transcription factor A (TFAM) knockdown (TFAM KD) was also used. MitoPark mice, a transgenic mouse model of PD that progressively develops mitochondrial dysfunction (Ekstrand and Galter, 2009), and the substantia nigra of postmortem human PD brains were then characterized for the accumulation of the identified H4 acetylation site. Our findings show significant elevation of H4K12ac deposition along with dysregulation of HAT and HDAC in both chemical-induced and CRISPR-mediated mitochondrial dysfunction in cell models as well as MitoPark mouse model and postmortem human PD brains, demonstrating the potentially important role of H4K12ac in the pathophysiology of PD.

## Materials and methods

### Chemicals and reagents

Fetal bovine serum (FBS), rotenone, pyridaben, and paraquat were purchased from Millipore Sigma. RPMI, L-glutamine, penicillin, streptomycin, IR-dye-tagged secondary antibodies, Hoechst nuclear stain, and other cell culture reagents were ordered from Invitrogen. The Bradford protein assay kit was purchased from Millipore, the Phalloidin antibody from Thermo Fisher Scientific, protease and phosphatase inhibitor cocktail from Life Technologies, acetylation inhibitor cocktail from Santa Cruz, cell viability assay (Cat# G3580) and chymotrypsin-like and caspase-like proteasome activity assay kits (Cat# G8661 and G8861) from Promega.

### AcetylScan proteomic profiling

The AcetylScan proteomic profiling followed our standard protocol (Huang et al., 2021). For each sample, at least 10 T175 flasks of N27 DAergic neuronal cells of the same cell passage were grown with a seeding density of 6 × 10^6^ cells/mL. Cells were collected and inspected by microscopy at 70% confluency. Following treatment with 1 μM rotenone for 3 h, cell harvesting proceeded until 10 flasks of cells had been harvested. Adherent cell cultures were washed with 10 mL of 4°C PBS. After pipetting off all PBS, 10 mL of urea lysis buffer (ULB) (20 mM HEPE pH 8.0, 9.0 M Pierce Sequanal grade urea, (Cat. No. #29700, Cell Signaling), 1 mM sodium orthovanadate (activated), 2.5 mM sodium pyrophosphate, and 1 mM beta-glycerol-phosphate) were added to flask#1 before scraping off the cells. For the remaining 9 flasks, steps were the same except the ULB came from the previous flask (e.g., flask #2 used ULB containing proteins from flask#1). Protein was digested and desalted using pre-conditioned C18 solid phase extraction. Peptides were then eluted from antibody beads with 50 μL of 0.15% trifluoroacetic acid (TFA), and completely dried by lyophilization. Next, peptides were resuspended and enriched by motif anti-Kac antibodies noncovalently coupled to protein A agarose beads and triple-washed with 1.5 mL buffer (Cat. No. #13416, Cell Signaling). After immunoprecipitation of beads, the resin was washed and peptides were eluted from it. Peptide fractions were analyzed by online nanoflow liquid chromatography tandem mass spectrometry (LC-MS/MS). Technical triplicates per sample were run. MS parameter settings were as follows: MS Run Time (96 min), MS1 Scan Range (300.0–1500.00), Top 20 MS/MS (Min Signal 500, Isolation Width 2.0, Normalized Coll. Energy 35.0, Activation-Q 0.250, Activation Time 20.0, Lock Mass 371.101237, Charge State Rejection Enabled, Charge State 1+ Rejected, Dynamic Exclusion Enabled, Repeat Count 1, Repeat Duration 35.0, Exclusion List Size 500, Exclusion Duration 40.0, Exclusion Mass Width Relative to Mass, Exclusion Mass Width 10 ppm). For data analysis, sequences were assigned to MS/MS spectra with Sorcerer for relative quantification. MS/MS spectra were evaluated using SEQUEST (J. K. Eng, 1994, J. Am. Soc. Mass Spectrom.) and the core platform from Cell Signaling. Searches were performed against the most recent update of the NCBI database with mass accuracy of ±50 ppm for precursor ions and 1 Da for productions. Results were filtered with mass accuracy of ±5 ppm on precursor ions and presence of the intended motif. Data were presented by fold change in acetyl-peptides, using a 2.5-fold cutoff to determine significant change.

### CRISPR/Cas9-based stable cell generation

The lentivirus-based CRIPSR/Cas9 TFAM-KD method conforms to our previous standard protocol (Huang et al., 2021). The TFAM-KO plasmid pLV-U6gRNA-Ef1aPuroCas9GFP-TFAM, with the TFAM-gRNA target sequence directed against the exon 1 sequence (CPR555e5e4099bf84.98), was acquired from Millipore Sigma. Based on manufacturer’s instructions, the lenti-CRISPR/Cas9 TFAM-KO plasmid and universal negative control lentivirus (U6-gRNA/CMV-Cas9-GFP) were respectively transfected into 293FT cells using the Mission Lentiviral Packaging Mix (Cat#SHP001, Millipore Sigma). The lentivirus was collected after 48 h from 293FT cells following transfection, then added to N27 cells for infection at an MOI of 100. After 24 h, puromycin (50 μg/mL) was added for stable cell selection.

### Animal studies

For this study, MitoPark (MP) transgenic (Tg), C57BL/6 and littermate control mice were bred and maintained under a 12-h light cycle in a climate-controlled mouse facility (22 ± 1°C) with food and water available *ad libitum*. MP Tg mice, with their TFAM conditionally knocked out through control of the dopamine transporter (DAT) as previously described, were provided by Dr. Nils-Goran Larsson of the Karolinska Institute in Stockholm (Ekstrand et al., 2007; Ekstrand and Galter, 2009). MP mice used in this study were obtained from the MP breeding colony at Iowa State University, genotyped to confirm their disease identity, and were further characterized using VersaMax for monitoring locomotor activity and RotaRod for testing coordination of movement. Equal numbers of male and female animals were allocated to experiment groups comprising 16 to 20-week age-matched C57BL/6 mice (TFAM^+/LoxP^: Dat^+/+^ from either MP or parental strain litters) or MP mice (Dat^+/Cre^: TFAM^LoxP/LoxP^).

### Human PD post-mortem brain samples

All human post-mortem samples were obtained from the brain banks at the Miller School of Medicine, University of Miami, FL, and the Banner Sun Health Research Institute, AZ (Please see our publication (Panicker et al., 2019) for detailed information on some of the human subjects included in this study). Samples were stored and distributed according to the applicable regulations and guidelines involving consent and the protection of human subjects and donor anonymity as described previously (Gordon et al., 2016; Huang et al., 2023). Freshly frozen SN cryosections and tissue blocks were clinically confirmed from post-mortem human PD patients and age-matched neurologically normal individuals. As to IHC experiments, cryosections were stained as described previously (Huang et al., 2021), but with slight modification. Antigen retrieval was done in citraconic anhydride buffer (0.05%, pH 7.4) at 80°C. For immunoblot experiments, tissue homogenates from freshly frozen tissue blocks were prepared at a final concentration of 1 mg/mL total protein from which 35 μg of total protein was directly used, or total histone was extracted from tissue blocks and 15–45 μg of total histone was used.

### RNA-seq library construction and sequencing

Following Illumina’s commercial protocol, total RNAs from samples were isolated and quality controlled. We used 2 μg RNA per sample in total as initial material for library construction. Poly-A-containing mRNA molecules were purified by using poly-T oligo-attached magnetic beads to fragment RNA samples into small pieces using divalent cations under elevated temperature. The cleaved RNA fragments were replicated into first strand cDNA using reverse transcriptase and random primers and replacing dTTP with dUTP, followed by second strand cDNA synthesis. These cDNA fragments with the addition of a single “A” base and subsequent ligation of the adapter were then purified and enriched with PCR to create the final cDNA library. RNA-seq was conducted on an Illumina HiSeq X10 platform, which generates single-end reads in the size of 150-bp. RNA-seq data were submitted to the GEO database (GSE180408).

The SN region of MitoParks was dissected and frozen immediately for RNA extraction. RNA extraction and cDNA library preparation followed the manufacture’s protocol provided by Quick Bio Inc., and Illumina next-generation platforms were used for sequencing. Quality control was performed to check the reads distribution in rRNA, exon, intron, intergenic regions, and the overall mapping rate in addition to ensuring the coverage was in the gene’s body region. FastQC software was used to check the original sequencing data quality. The trimmed mean of *M*-values method in the edgeR package was used to normalize gene expression. EdgeR was applied to perform the differential gene analysis and the cutoffs were *p* < 0.05 and fold change >1.5. Of note, low expression genes were filtered. Genes with detectable expression (cpm >1) in at least one group were kept. The reads were first mapped to the latest UCSC transcript set using Bowtie2 version 2.1.0 (Langmead and Salzberg, 2012) and the gene expression level was estimated using RSEM v1.2.15. (Li and Dewey, 2011) Differentially expressed genes were identified using the edgeR program (Robinson et al., 2010). Genes showing altered expression with *p* < 0.05 and more than 1.5-fold changes were considered differentially expressed. GOseq (Young et al., 2010) was used to perform the GO enrichment analysis, and Kobas (Xie et al., 2011) was used to perform the pathway analysis.

### Whole-genome gene expression analysis

Data analysis was performed similar to Huang et al. (2021). After removing the adapter sequences from the raw sequencing data, the individual libraries were converted to the FASTQ format. Sequence reads were aligned to the specific genome of the corresponding species with HISAT2 (v2.1.0) and the resulting alignment files were reconstructed with the EdgeR3 package. RefSeq database (Build 37.3) was chosen as the annotation references for mRNA analysis. The read counts of each transcript were normalized to the length of the individual transcript and to the total mapped fragment counts in each sample and expressed as reads per kilobase of exon per million fragments mapped (RPKM) of mRNAs in each sample. The mRNA differential expression analysis was applied to treatment and control groups. An adjusted *p*-value <0.05 (student’s *t*-test with Benjamini–Hochberg FDR adjustment) was used as the cut-off to denote statistical significance. Differential expression genes (DEGs) were analyzed by enrichment analysis to detect over-represented functional terms present in the genomic background. GO and KEGG pathway analyses were performed using the DAVID Bioinformatics Resources 6.8 (Kanehisa and Goto, 2000; Huang et al., 2009; Valentine et al., 2019).

### Mitochondrial bioenergetics analysis

Following our standard protocol reported in Huang et al. (2021), Seahorse XFe24 analyzer was used for the Mito Stress test to measure mitochondrial OCR and ECAR. For this study, 0.75 μM oligomycin, 1 μM carbonyl cyanide p-trifluoromethoxyphenylhydrazone, and 0.5 μM rotenone-antimycin in serum-free medium were used.

### SYBR green RT-qPCR

RNA from samples was isolated according to the manufacturer’s protocol. In brief, cells were lysed in 1 mL TRIzol reagent, incubated for 5 min at room temperature (RT), mixed with 0.2 mL chloroform, and incubated for 2 min. For tissues, the protocol has a slight modification specifying that homogenization be performed after adding TRIzol. Following centrifugation, the top layer containing RNA was transferred to a new tube containing 0.7 mL isopropanol for a 15-min incubation. After centrifugation again, RNA pellets were washed with 75% ethanol, air-dried, and dissolved in water. The High-Capacity cDNA Synthesis kit from Applied Biosystems (#4368814) was applied for cDNA synthesis following the manufacturer’s protocol. For normalization of each sample in RT-qPCR, the 18S rRNA gene of the corresponding species (Cat. No. PPM57735E, Qiagen) was used as the housekeeping gene. According to manufacturer’s guidelines, quality controls were performed by running dissociation curves and melting curves to ensure single amplicon peaks without any non-specific amplicons. Quantitative PCR was performed on qRT^2^PCR SYBR Green Mastermix (Agilent). Fold change in gene expression was determined using the ΔΔC_t_ threshold cycle (C_t_) method.

### Proteasome activity assay

Following the manufacture’s protocol, the reagent was equilibrated at RT for 30 min and added to cells to make 50 μL per well. After mixing and incubation for 10 min, the plate was read by a luminometer.

### Immunoblotting

Brain tissues and cell culture lysates were prepared using modified RIPA buffer and were normalized for equal amounts of protein. Equal amounts of protein (6–10 μg for nuclear lysates, 10–25 μg for cell lysates and 30–40 μg for tissue lysates) were loaded for each sample and separated on either commercial or 15% SDS-PAGE gels depending on the molecular weight of the target protein. After separation, proteins were transferred to a nitrocellulose membrane and the nonspecific binding sites were blocked for 1 h using a blocking buffer specifically formulated for fluorescent Western blotting (Rockland Immunochemicals). Membranes were then probed with the respective primary antibodies for 3 h at RT or overnight at 4°C. Incubated membranes were washed seven times with PBS containing 0.05% Tween-20, and then secondary IR-800 conjugated goat anti-mouse IgG (LI-COR, 1:20000) and IR-700 conjugated goat anti-rabbit IgG (LI-COR 1:20000) antibodies were used for detection with the Odyssey IR imaging system (LI-COR). The primary antibodies used were H4K12ac, HDAC1, HDAC2, HDAC3, and HDAC4 (Cell Signaling, 1:1000). Antibodies for β-actin and H3 were used as loading controls. Antibodies against Lamin B were used as the loading control for nuclear lysates.

### HAT activity assay

The manufacturer’s protocol was followed. In brief, each plate map was designed with positive controls, negative controls, and background wells. We added β-mercaptoethanol for the standard curves, p300 catalytic domain for positive control, and anacardic acid for inhibitor wells. After adding acetyl-CoA, plates were incubated with H4 peptide substrate for 30 min, a stop solution, and a final developing solution before being read with excitation at 360 nm and emission at 450 nm. As to background wells, the stop solution was added before the substrate.

### Immunocytochemistry

For ICC, 4% paraformaldehyde (PFA) was used to fix N27 DAergic neuronal cells. After a double-wash, fixed cells were blocked and then incubated in primary antibodies following the manufacturer’s protocol as previously published (Zhang et al., 2022). After primary antibody (H4K12ac, HDAC1, HDAC2, HDAC3, HDAC4, 1:750, Cell Signaling) incubation, cells were washed with PBS and incubated in Alexa dye-conjugated secondary antibody. Next, cells were washed and mounted on slides using Fluoromount aqueous mounting medium (Sigma). Cells were visualized using an inverted fluorescence microscope (Keyence BZ-X800).

### Cell viability assay

The manufacture’s protocol was followed with a minor modification. Cells were seeded into a 96-well plate overnight. Then, 10 μL per well of the reagent was added to the plate, after which the plate was incubated at 37°C for 1 h in a humidified, 5% CO_2_ atmosphere. Data were recorded by a plate reader at 490 nm and at 650 nm for background subtraction.

### Nuclear and cytoplasmic fractionation

Nuclear and cytoplasmic fractions were performed using the NE-PER Kit (Thermo Scientific) as per the commercial protocol. Briefly, 5 × 10^6^ cells were harvested. CER1 reagent (150–200 μL) was used per sample to extract the cytoplasmic fraction and 45 μL of NER reagent was used to extract the nuclear fraction.

### Immunohistochemistry and immunofluorescence studies

Immunohistochemistry (IHC) was performed on sections from the brain regions of interest following standard protocol in our lab. Briefly, mice were anesthetized with a mixture of 200 mg/kg ketamine and 20 mg/kg xylazine and then perfused transcardially with freshly prepared 4% PFA. Extracted brains were post-fixed in 4% PFA for 48 h and 30-μm sections were cut using a freezing microtome (Leica Microsystems). Antigen retrieval was performed in sodium citrate buffer (10 mM, pH 8.5) for 30 min at 90°C. PBS-washed sections were blocked with PBS containing 2% BSA, 0.2% Triton X-100 and 0.05% Tween-20 for 1 h at RT before being incubated with primary antibodies overnight at 4°C and washed seven times in PBS on a Belly Dancer Shaker (SPI supplies). Sections were incubated with Alexa 488, 555, and 633 dye-conjugated secondary antibodies for 75 min at RT and their cell nuclei were stained with Hoechst dye. Samples were then washed several times in PBS and coverslips were mounted with Fluoromount medium on glass slides for visualization. Staining intensity was imaged by assessing the natural fluorescence using a Keyence microscope.

### Confocal imaging and Z stack image acquisition and analysis

Confocal imaging was performed at the Iowa State University’s Microscopy Facility using the Leica DMIRE2 confocal microscope with the 63X and 40X oil objectives and Leica Confocal Software. One optical series covered 11–13 optical slices of 0.5-μm thickness each. Imaris software was used to analyze Z stack images. The surface reconstruction wizard in Imaris was used to generate 3D reconstructions of stacks for easier viewing of microglial-dopaminergic contacts. IHC on human sections was performed as described above, but with some modifications. Antigen retrieval was done overnight in sodium citrate buffer (10 mM, pH 8.5) at 4°C prior to the 90°C step.

### Study approval

Iowa State University’s (ISU, Ames, IA, United States) laboratory animal facility is fully accredited by the Association for Assessment and Accreditation of Laboratory Animal Care International (AAALAC), and all procedures involving animal handling were approved by the Institutional Animal Care and Use Committee (IACUC) at ISU. For human samples, since deidentified post-mortem human brain tissues were obtained from approved national brain banks, Institutional Review Board (IRB) approval from ISU was not required.

### Statistical analysis

Data analysis was performed using GraphPad Prism 7.0. Normally distributed raw data were analyzed with either student’s *t*-test (2-group comparisons) or one-way ANOVA (≥3-group comparisons) with Tukey *post hoc* test unless otherwise mentioned. Statistically significant differences were denoted as ^*^p ≤ 0.05, ^**^p < 0.01, and ^***^p < 0.001.

## Results

### H4K12ac is a key epigenetic marker in response to mitochondrial dysfunction in dopaminergic neuronal cells

**Figure 1:**
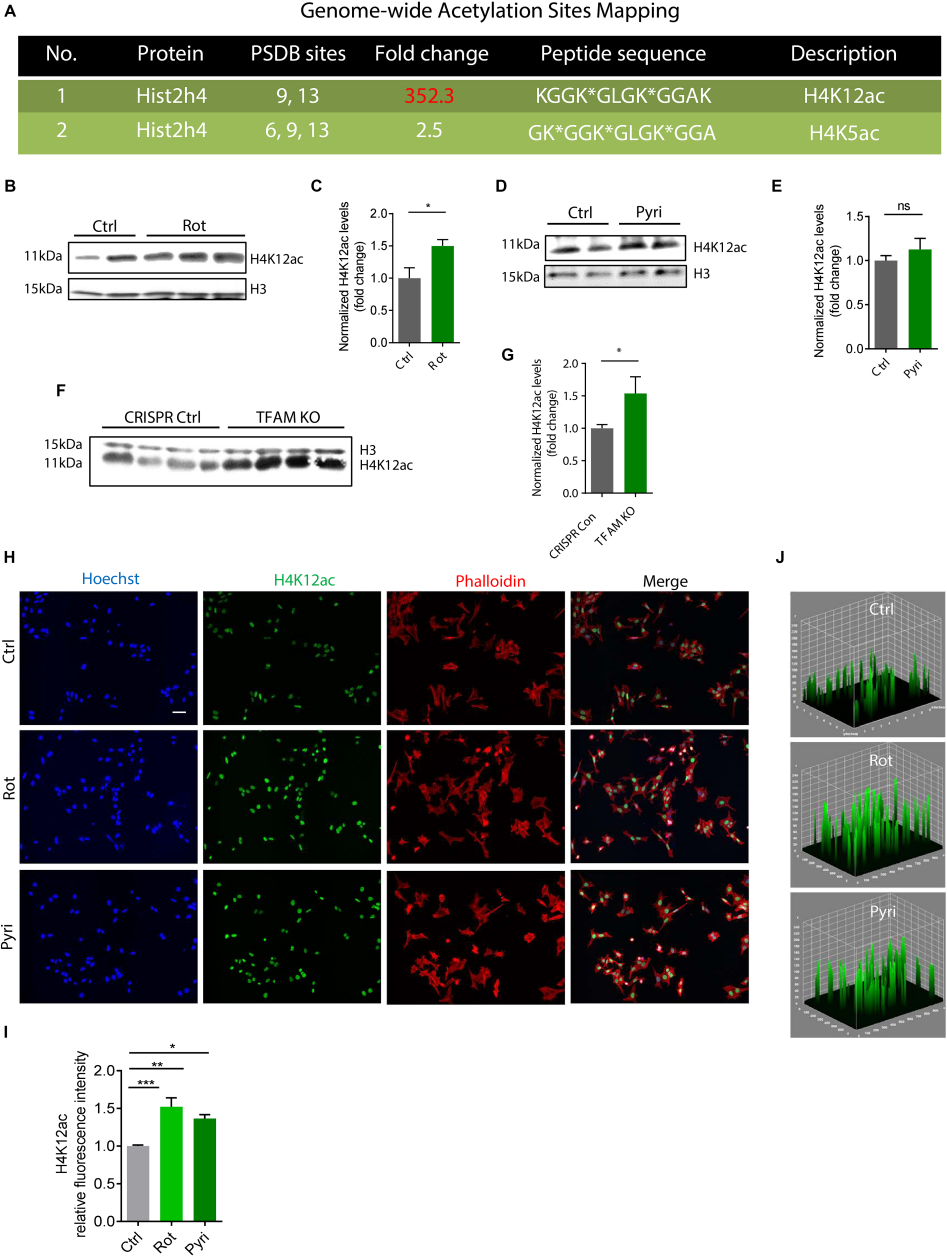
H4K12ac deposition elevated in rotenone (Rot)-exposed, pyridaben (Pyri)-exposed, and TFAM-KO DAergic neuronal N27 cell models of PD. **(A)** Genome-wide acetylation site mapping of core histone H4 of rotenone-exposed N27 cells using LC/MS coupled with novel anti-acetyl-lysine antibodies and an optimized acetylome workflow. In the AcetylScan, only fold changes >2.5-fold were tracked with asterisks denoting acetylation sites (*n* = 2). Peptide sequences of H4K12ac and H4K5ac are shown in histone cluster 2 H4. **(B)** Representative immunoblots and **(C)** their quantification for H4K12ac from Rot-treated N27 cells (*n* = 4–6). **(D)** Representative immunoblots for H4K12ac and **(E)** their quantification from Pyri-treated N27 cells (*n* = 6–8). **(F)** Representative immunoblots and **(G)** their quantification from TFAM-KO N27 cells. **(H)** Immunocytochemistry for H4K12ac (green) from Rot-treated, Pyri-treated, and TFAM-KO N27 cells, with the nucleus stained with Hoechst (blue) and actin filaments with Phalloidin (red). Independent experiments were repeated at least four times. Scale bar, 50 μm. **(I)** Keyence BZ-X800 analysis of fluorescence intensity of H4K12ac. **(J)** 3D surface plot analysis of H4K12ac in Rot-treated and Pyri-treated N27s. Bar graphs show mean ± s.e.m. of one-way ANOVA followed by Tukey’s *post hoc* test. ns, not significant; ^*^p < 0.05, ^**^p < 0.01, and ^***^p < 0.001.

In the first set of experiments, we used a mitochondrial complex I inhibitor, rotenone, to challenge DAergic neuronal N27 cells in a low-dose treatment paradigm (1 μM for 3 h) and mapped the acetylation profile of core histone H4 using the immunoaffinity-based AcetylScan proteomic method (Svinkina et al., 2015). Of many H4 acetyl sites, the K12 was shown to have the highest acetylation level following rotenone treatment, increasing ~350-fold over the control (Figure 1A). This K12ac site is located at histone cluster 2, H4. Its peak corresponds with the acetylation of other sites, that is, K5 and K8, which possibly assist K12ac in its functional role. Since we found that K12 acetylation displayed the highest increase following rotenone treatment, we hypothesized that it was a critical histone modification site in response to environmental neurotoxic pesticide exposure. To validate the proteomic results, we used the same cell lysate from the AcetylScan experiment to check the levels of H4K12ac and H4K5ac by immunoblotting. In line with Figure 1A, both H4K5ac and H4K12ac increased accordingly (Supplementary Figure S1). Significantly increased levels of H4K12 also occurred in the rotenone-insulted histone extracts (Figures 1B,C). To further confirm that H4K12 hyperacetylation is induced by mitochondrial dysfunction, we applied another mitochondrial complex I inhibitor, pyridaben, to N27 DAergic cells. Pyridaben can impair mitochondrial electron transport function and bring about an increase in the permeability transition and reactive oxygen species (ROS) as rotenone does. We treated N27 cells with pyridaben using a similar low-dose paradigm (3 μM for 3 h) and checked H4K5 and K12 acetylation. Consistently, our results show significantly elevated deposition of H4K5ac and H4K12ac in pyridaben-exposed cells (Supplementary Figure S2; Figures 1D,E). Although acetylation of K5 and K12 sites both upregulated, considering that K5 is much less inducible (Figure 1A; Supplementary Figures S1, S2), K12 should be the primary site for mitochondrial dysfunction-induced H4 hyperacetylation. In addition, we knocked down (KD) the key regulator of mitochondrial genome transcription and mitochondrial biogenesis, TFAM, in N27 cells (Langley et al., 2018). Similarly, immunoblots of total histones isolated from TFAM-KD N27 cells demonstrate an increase in the H4K12ac levels (Figures 1F,G). Our ICC results (Figures 1H–J) of rotenone-exposed, pyridaben-insulted, and TFAM-KD N27 cells further validated the immunoblot results, showing a higher density of brighter green peaks in treatments than controls indicating a higher expression level of H4K14ac (Figure 1J, a 3D plot of H4K12ac stained in green showing K12ac expression peaks). Collectively, our findings suggest H4K12ac acts as a sensitive epigenetic marker in response to acute, low-dose environmental neurotoxic pesticide exposure and mediates the signaling cascade of mitochondrial dysfunction.

### Paraquat exposure increases H4K12ac deposition in N27 cells

**Figure 2:**
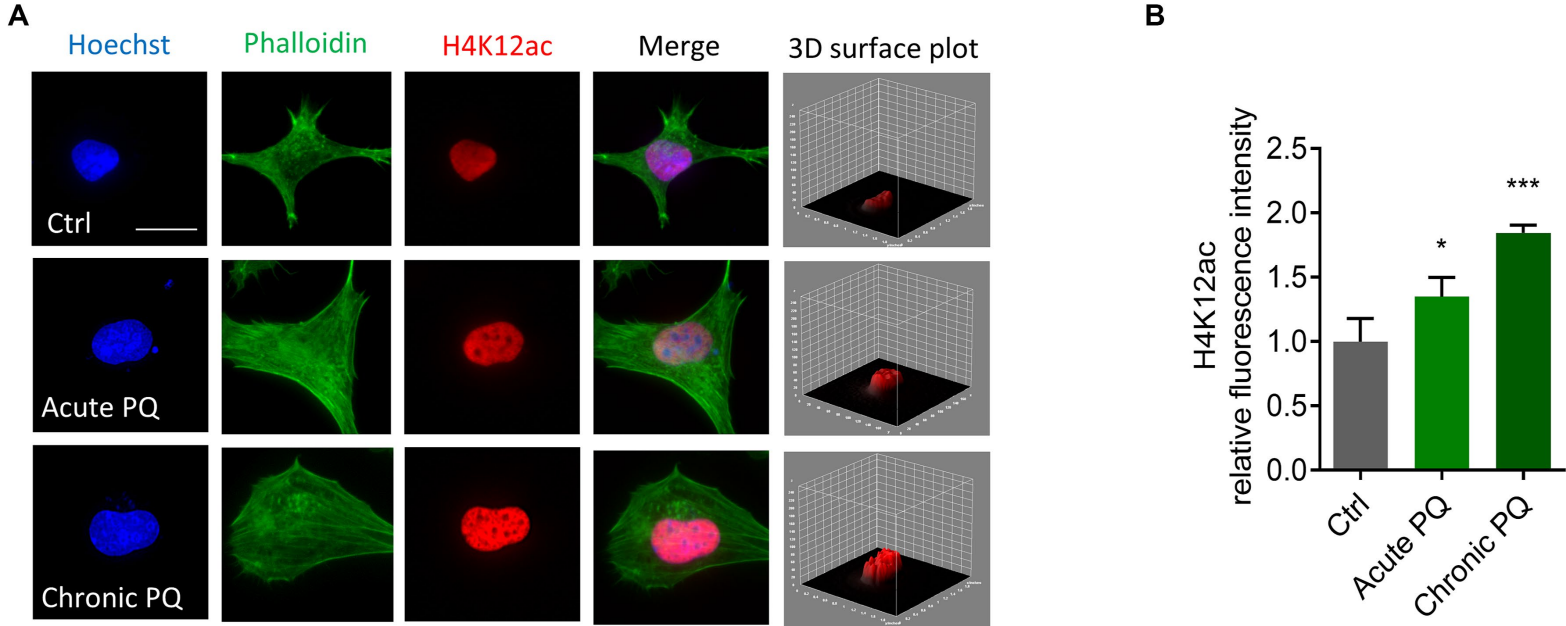
H4K12ac deposition increased in acutely and chronically paraquat (PQ)-exposed N27 cells. **(A)** Immunocytochemistry for H4K12ac (red) and its 3D surface plot analysis from acute and chronic PQ treatments in N27 cells, with the nucleus stained with Hoechst (blue) and actin filaments with Phalloidin (green). Independent experiments were repeated three times. Scale bar, 20 μm. **(B)** Keyence BZ-X800 analysis of fluorescence intensity of H4K12ac. Bar graphs show mean ± s.e.m. of one-way ANOVA followed by Tukey’s *post hoc* test. ns, not significant; ^*^p < 0.05 and ^***^p < 0.001.

Our previous studies found that the neurotoxic pesticide paraquat induces core histone H3 hyperacetylation without causing significant acetylation change in total H4 (Song et al., 2011). As such, we studied the acetylation status of a specific site, H4K12, following paraquat exposure. The widely used pesticide paraquat shares structural similarities with the DAergic neurotoxicant MPTP (1-methyl-1,2,3,6-tetrahydropyridine), a chemical for inducing Parkinsonian syndrome in laboratory animals and has been linked to the etiologic factors of PD (Li et al., 2005; Dinis-Oliveira et al., 2006; Tanner et al., 2011; Vaccari et al., 2017). We insulted N27 cells with paraquat using both a normal-dose acute treatment paradigm (400 μM for 6 h) and a low-dose chronic paradigm (100 μM for 24 h). ICC results demonstrate a marked hyperacetylation in H4K12 for both acute and chronic treatments (Figure 2). Notably, H4K12ac appears more sensitive to the low-dose chronic paraquat treatment.

### H4K12ac linked to DAergic neuronal degeneration in MitoPark mice

**Figure 3:**
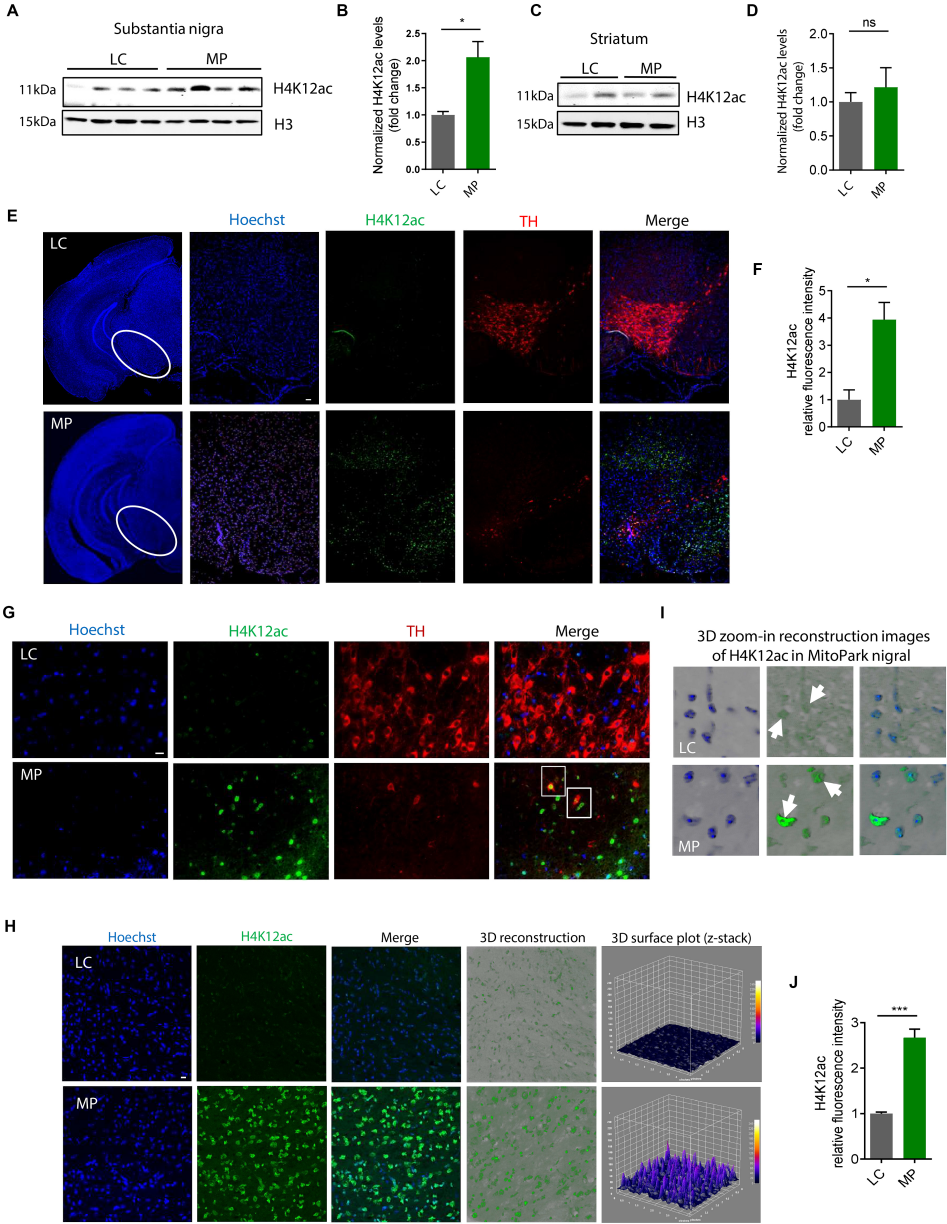
H4K12ac deposition increased in the SN of MitoPark mice. **(A)** Representative immunoblots and **(B)** their quantification from SN of MPs and LCs. Unpaired test followed by Welch’s correction (*n* = 4). **(C)** Representative immunoblots and **(D)** their quantification from striatum of MPs and LCs. **(E)** Immunohistochemistry using 7-μm thick paraffin-embedded sections with H4K12ac in green, TH in red, and nucleus in blue (*n* = 3). Scale bar, 50 μm. **(F)** Their quantification and **(G)** their zoomed-in images. **(H)** Confocal images, 3D reconstruction images, and 3D surface plot analysis of 30-μm free-floating sections from same-aged mice with H4K12ac in green and nucleus in blue. Scale bar, 5 μm. **(I)** Zoomed-in images of **(H)**. Arrows denote H4K12ac. **(J)** Their quantification for H4K12ac (*n* = 3–4). Bar graphs show mean ± s.e.m. of unpaired two-tailed *t* tests. ns, not significant; ^*^p < 0.05 and ^***^p < 0.001.

Having shown that H4K12ac mediates the signaling cascade of mitochondrial dysfunction, we then extended these findings *in vivo* to the progressively neurodegenerative MitoPark mouse model. Our prior data show that the expression levels of the total H3 itself increased in both MitoPark mice and postmortem tissues of PD patients compared to the non-disease groups (data not shown), in alignment with another recently published human PD study (Toker et al., 2019), therefore we used both β-actin and H3 as loading controls in this study. Immunoblotting shows H4K12ac significantly increases in the SN of 16–19-week MitoParks (Figures 3A,B), instead of the striatum (Figures 3C,D), indicating a possible link between H4K12ac and nigral DAergic neurodegeneration.

To look closer at the co-occurrence of H4K12ac and DAergic neuronal cell death, we performed IHC co-staining with H4K12ac (green) and TH (red) on SN sections from MitoParks and littermate controls at an age (16–18-week) when motor deficits and cognitive dysfunction have emerged and DAergic neurons are degenerating. In MitoParks (Figure 3E, red channel), a certain number of DAergic neurons had degraded, while some other neurons were in the process of degrading. H4K12ac deposition (Figure 3E, green channel) increased dramatically at the relatively later period of the degradation process, and its presence lasts even after some DAergic neurons have completely degraded. Automated quantification of H4K12ac fluorescence intensity (Figure 3F) via BZ-X800 analyzer confirms that H4K12ac deposition increased significantly in the SN of mitochondria-impaired MitoParks, supporting our *in vitro* data (Figures 1, 2). The colocalization of H4K12ac and DAergic neurons (Figure 3G, white rectangles) demonstrates that elevated H4K12ac deposition occurred within DAergic neurons. Significantly increased H4K12ac deposition is further evidenced by 3D surface plot analyses in the nigral MitoParks (Figures 3H–J). Taken together, these data demonstrate that the H4K12ac marker possibly correlates with DAergic neuronal degeneration.

### Elevated HK12ac deposition in the SN of postmortem human PD brains

**Figure 4:**
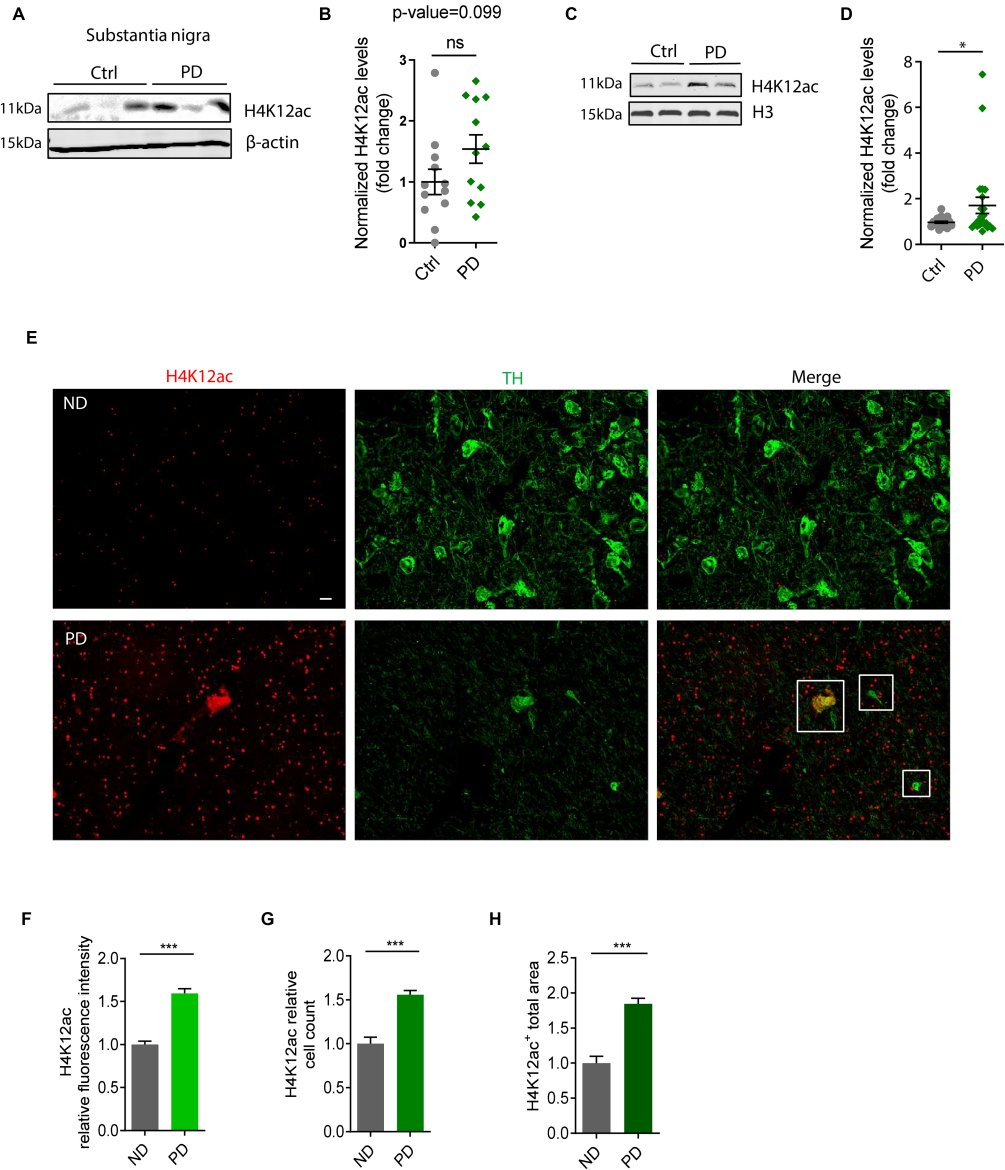
H4K12ac deposition elevated in the substantia nigra (SN) of human PD. **(A)** Representative immunoblots showing increased H4K12ac in SN lysate of human PD relative to age-matched controls. **(B)** Densitometric analysis of immunoblots (*n* = 12, *p* = 0.099) with normalized β-actin as the internal control. **(C)** Representative immunoblots and **(D)** their quantification of histone extracts in SN lysate of human PD and age-matched controls with histone H3 as the internal control (*n* = 21–23). **(E)** Immunohistochemistry in SN sections of human PD and age-matched non-disease (ND) controls (*n* = 3–4) captured by confocal imaging. Squares denote the colocalization between H4K12ac (red) and TH (green). Scale bar for **(E)**, 10 μm. **(F)** Quantified fluorescence area, **(G)** count of cells, and **(H)** fluorescence intensity for H4K12ac. Five regions of each human SN section were randomly selected. Bar graphs show mean ± s.e.m. of unpaired two-tailed *t* tests. ns, not significant; ^*^p < 0.05 and ^***^p < 0.001.

To highlight the significance of our *in vitro* and *in vivo* findings on H4K12ac deposition (Figures 1–3), we tested their clinical relevance in PD patients. Samples of the SN from brains collected postmortem from PD patients and age-matched controls were immunoblotted with the antibody against H4K12ac. Both whole SN lysates and histone extracts were checked. The whole SN lysates of human PDs display an elevated trend of H4K12ac accumulation compared to age-matched controls (*n* = 12, *p*-value = 0.099) (Figures 4A,B). Supporting the stimulated H4K12ac in PD patients, immunoblotting of nuclear histone extracts from more SN samples of PD and age-matched controls (*n* = 21–23) confirms the significantly increased accumulation of H4K12ac in the nigral of PD patients (Figures 4C,D).

To further confirm the H4K12ac marks in human PD nigral sections and the possible link between H4K12ac and DAergic neurodegeneration, we double-immunostained human nigral sections with H4K12ac (red) and TH (green) antibodies. IHC analysis displays a sharply increased number of H4K12ac marks in the human PD SN and its colocalization with surviving DAergic neurons (Figure 4E), consistent with the IHC results of MitoParks in Figures 3E–H. Enhanced H4K12ac immunoreactivity in PD was also verified by quantifications of H4K12ac fluorescence intensity, H4K12ac^+^ cell count, and H4K12ac^+^ total area (Figures 4F–H). To sum up, the human SN data add clinical relevance to our findings from experimental PD and highlight the translational potential of anti-H4K12ac therapy for PD.

### HAT/HDAC imbalance and proteasome impairment modulate H4K12 hyperacetylation

**Figure 5:**
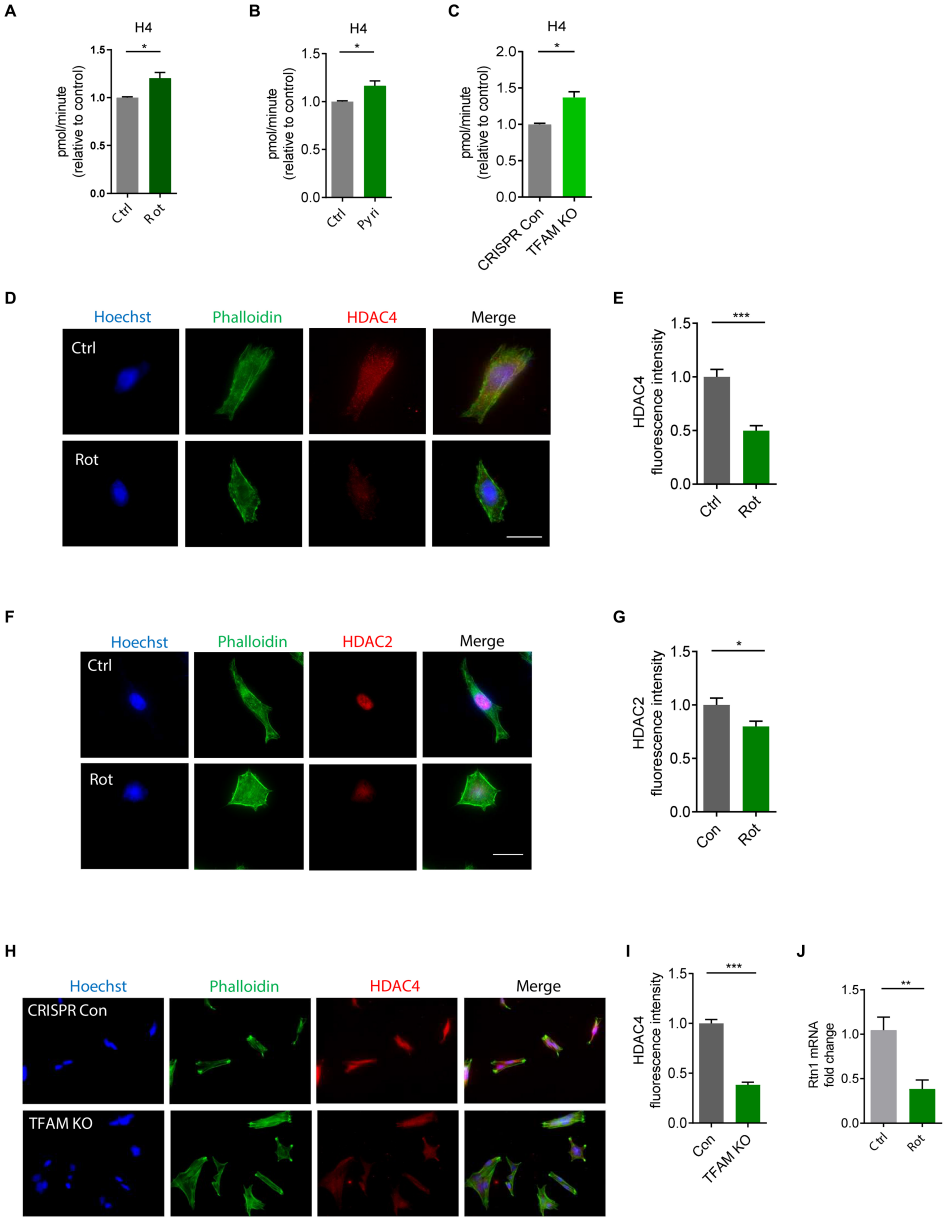
HAT/HDAC imbalances in rotenone (Rot)-exposed, pyridaben (Pyri)-exposed, and TFAM-KO N27 cells. **(A)** Evaluation of HAT activity using H4 peptide as the substrate in Rot-treated N27 cells with *n* = 5–6, **(B)** Pyri-treated N27 cells with *n* = 6–8, and **(C)** TFAM-KO N27 cells with *n* = 4. **(D)** Immunocytochemistry for HDAC4 (red) and its quantification **(E)** from Rot-treated N27 cells (*n* = 6–7) with the nucleus stained with Hoechst (blue) and actin filaments with Phalloidin (green). **(F)** Immunocytochemistry for HDAC2 (red) and **(G)** its quantification from Rot-treated N27 cells (*n* = 7). **(H)** Immunocytochemistry for HDAC4 (red) and **(I)** its quantification from TFAM-KO N27 cells *n* = 5. **(J)** RT-qPCR of the HDAC-controlled gene *Rtn1* in Rot-treated N27 cells. For immunocytochemistry, independent experiments were repeated three times. Bar graphs show mean ± s.e.m. of unpaired two-tailed *t* tests. ns, not significant; ^*^p < 0.05, ^**^p < 0.01, and ^***^p < 0.001.

**Figure 6:**
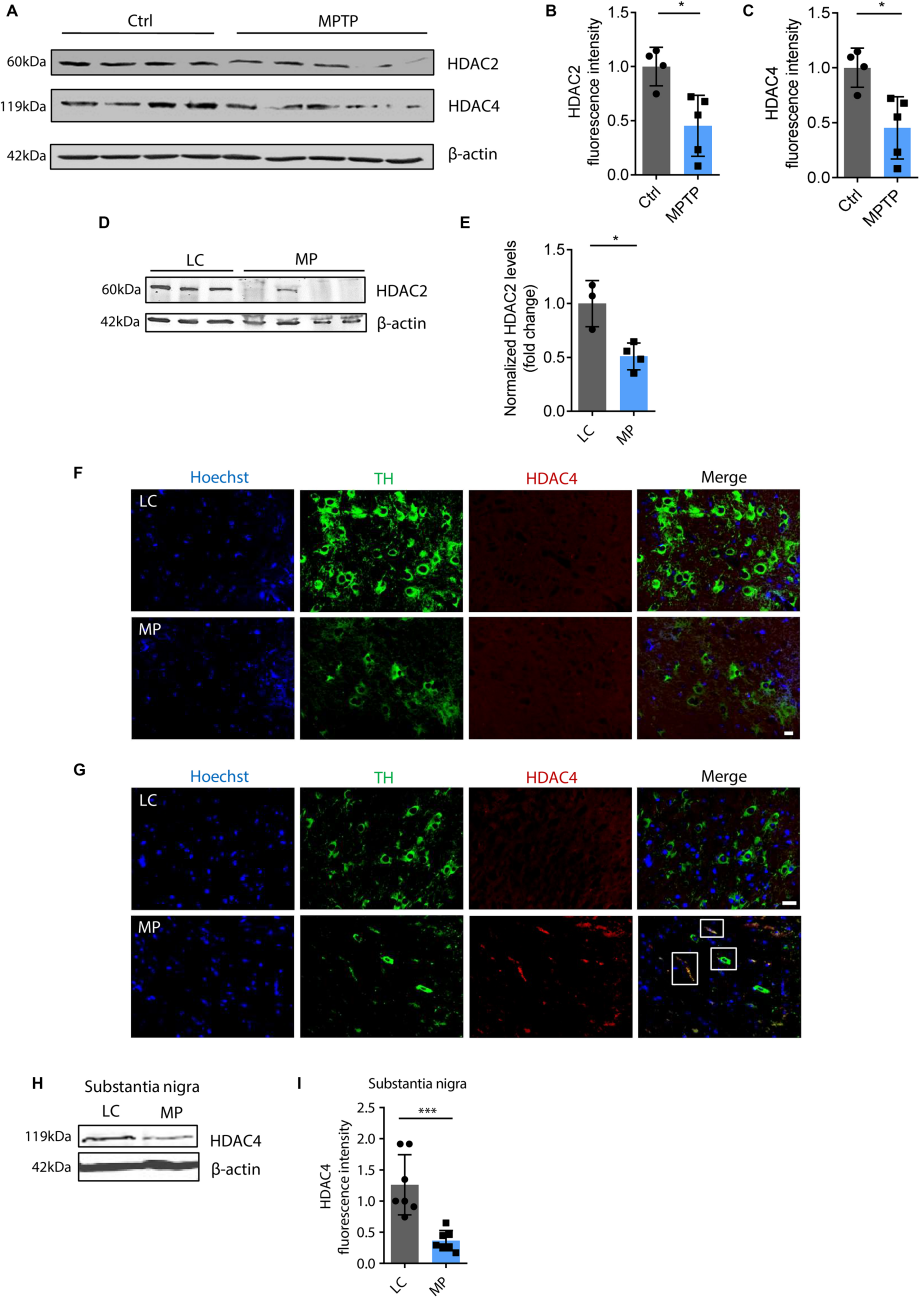
HDAC4 and HDAC2 decreased in MPTP-insulted mice and MitoParks. **(A)** Immunoblots for HDAC2 and HDAC4 in MPTP-treated C57BL/6 mice (*n* = 4–5) and **(B,C)** their respective densitometric analyses. **(D)** Representative immunoblots for HDAC4 in SN of 16–18-week littermate controls (LC) and MitoPark (MP) mice coupled with **(E)** their densitometric analyse (*n* = 7). **(F)** Immunohistochemistry for HDAC4 in SN of 14-week and **(G)** 16–17-week LC and MP mice (*n* = 4). **(H)** Representative immunoblots for HDAC4 in SN of 18 ~ 20-week LC and MP mice and **(I)** their quantification (*n* = 7). Error bars show mean ± s.e.m. of unpaired two-tailed *t* tests. ns, not significant; ^*^p < 0.05 and ^***^p < 0.001.

The functional interplay between HATs and HDACs regulates the balance of histone acetylation. The significant role of HAT/HDAC homeostasis is highlighted by an ongoing investigation of a new pharmacological strategy using an HDAC inhibitor (ClinicalTrials.gov number, NCT02046434) for α-synuclein clearance from the brain and a funded study on the modulation of HDAC4 in patients carrying either the LRRK2, GBA, or SNCA mutation as a PD treatment sponsored by Michael J. Fox Foundation. Our prior study found that paraquat represses HDAC activity in N27 DAergic cells (Song et al., 2011). As such, we proposed that an HAT/HDAC imbalance occurs following mitochondrial dysfunction, which may account for the observed H4K12 hyperacetylation. To examine HAT/HDAC homeostasis, we first detected the total HAT activity with an H4 peptide as the substrate. The results reveal significantly increased HAT activity in rotenone-exposed, pyridaben-exposed, and TFAM-KD N27 cells (Figures 5A–C). Next, we performed ICC to explore HDACs dysfunction in N27 cells. Among HDAC isoforms, HDAC2 and HDAC4 significantly decreased in rotenone-exposed N27 cells (Figures 5D–G), while HDAC1 and HDAC3 showed negligible change (data not shown). Significantly reduced HDAC4 immunoreactivity was also observed in stable TFAM-KD N27 cells (Figures 5H,I). Consistent with these results, immunoblotting of HDAC2 and HDAC4 as well as ICC of HDAC2 reveal a similar pattern in TFAM-KD cells (Supplementary Figure S3). However, HDAC1 showed no significant change in rotenone-treated N27 cells (Supplementary Figures S4A,B). These results demonstrate the tilting of HAT/HDAC homeostasis in our cell models of PD. Recently, a publication studied iPSC-derived DAergic neurons from patients with GBA-N370S risk variant and identified HDAC4 as an upstream regulator of PD progression. Treatment with HDAC4-modulating compounds can correct HDAC4-controlled gene expression and attenuate ER stress as well as autophagic and lysosomal perturbations (Lang et al., 2019). This testifies to the potential of modulating HDAC4 as an epigenetic-based therapy. To understand the downstream gene expression modulated by the transcriptional repressor HDAC4 in our mitochondria-stressed cell model of PD, the core set of four HDAC4-controlled genes, including *Tspan7*, *Atp1a3*, *Rtn1*, and *Prkcb* (Lang et al., 2019), was analyzed by RT-qPCR. *Rtn1*, a gene involved in membrane trafficking and regarded as a marker for neurological diseases, was downregulated in rotenone-exposed N27 cells, possibly mediated by HDAC4 downregulation (Figure 5J). In our *in vivo* studies, HDAC2 and HDAC4 protein levels were reduced in the SN of MPTP-treated C57BL/6 mice (Figures 6A–C), whereas no significant change was observed in HDAC1 (Supplementary Figures S4C,D). Similar to MPTP-treated C57BL/6 mice, HDAC2 expression level decreased in the SN of 18–20-week MitoParks (Figures 6D,E). Moreover, IHC imaging in the SN of 16–17-week MitoParks (Figure 6F) shows an increased level of HDAC4 in comparison to 14-week MitoParks (Figure 6G). In 18–20-week MitoParks, the expression level of HDAC4 decreased in the SN (Figures 6H,I).

**Figure 7:**
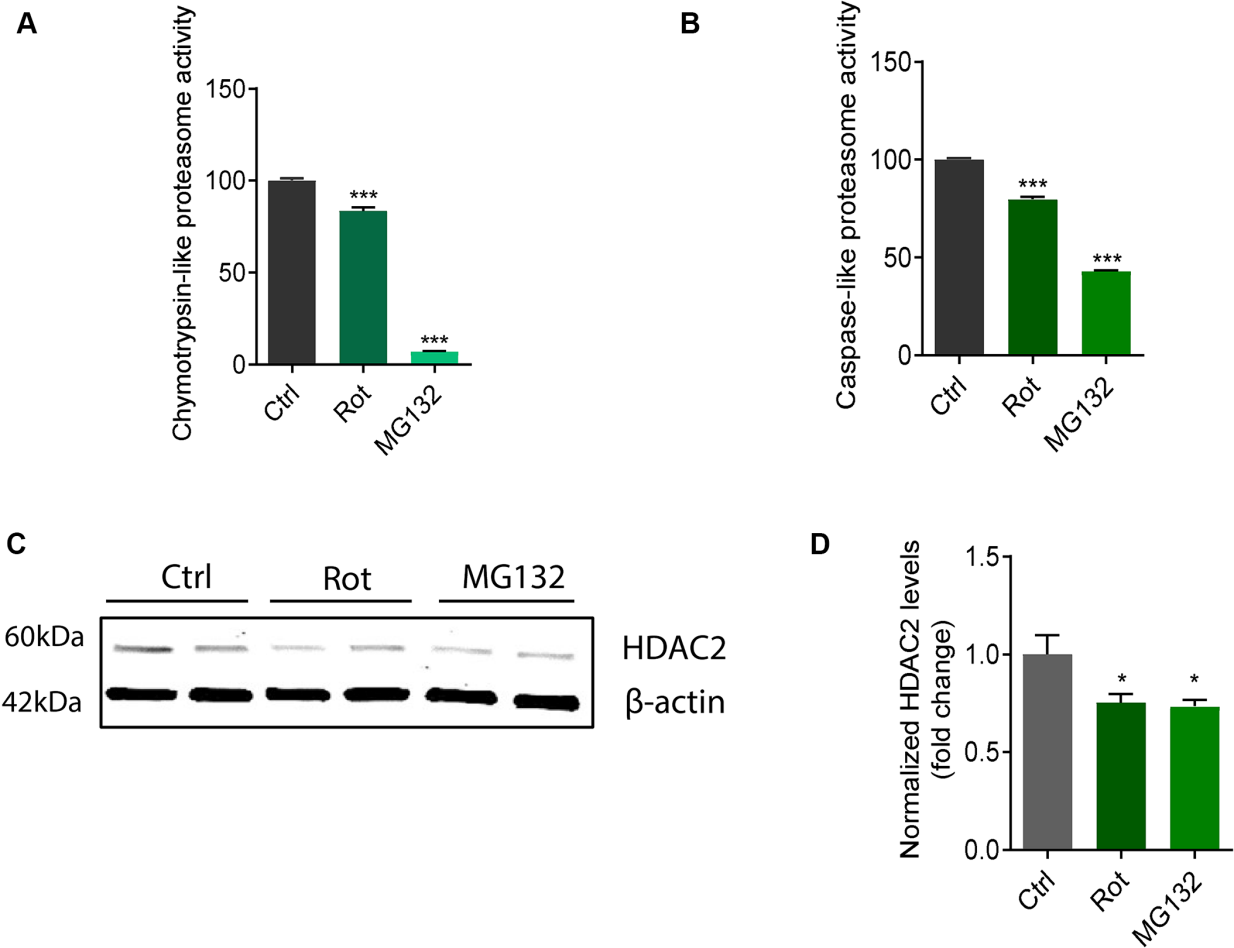
Rotenone (Rot) impaired chymotrypsin-like and caspase-like proteasome activity in N27s and HDAC2 decreased in MG132-exposed N27s. **(A)** Chymotrypsin-like and **(B)** caspase-like measurements of proteasome activity (*n* = 6). **(C)** Representative immunoblots for HDAC2 in Rot-treated and MG132-treated N27 cells coupled with **(D)** their densitometric analysis (*n* = 4–5). Error bars show mean ± s.e.m. of unpaired two-tailed *t* tests. ns, not significant; ^*^p < 0.05 and ^***^p < 0.001.

Dysfunctional mitochondria can result in diminished ubiquitin-proteasome system (UPS) activity due to cellular bioenergy deficits (Segref et al., 2014; Karbowski et al., 2022). Many UPS components contain mitochondrial targeting sequences interacting with mitochondrial proteins (Lehmann et al., 2016). Further, accumulative evidence suggests impaired UPS in PD leads to aberrant protein accumulation/aggregation and cytotoxicity (Sun et al., 2007; Lehmann et al., 2016). Rotenone and the proteasome inhibitor MG132 were reported to impair proteasome activity and perturb protein homeostasis (Betarbet et al., 2006; Sun et al., 2006). Therefore, we examined proteasome activity in rotenone-exposed and MG132-exposed N27 cells. Following rotenone and MG132 treatments, proteasome activity was significantly reduced specifically in caspase-like and chymotrypsin-like proteasome activity (Figures 7A,B). Interestingly, both rotenone and MG132 treatment decreased HDAC2 protein expression in N27 cells (Figures 7C,D), suggesting that mitochondrial stressor-induced HDAC2 downregulation is mediated at least partly by proteome impairment. Like rotenone, the functional consequences of the mitochondrial impairment attributed to MPP^+^ treatment in N27 cells emerged as reductions in basal respiration rate, spare respiratory capacity, proton leak, and ATP production as revealed by the extracellular flux analyses (Supplementary Figures S5A–C). A cell energy phenotype analysis demonstrates that MPP^+^ also stressed metabolic activity as measured by the OCR and ECAR/glycolysis (Supplementary Figure S5D).

To summarize, mitochondrial dysfunction perturbs protein homeostasis, UPS and proteasome functionality, and alters the HAT/HDAC ratio, all of which may contribute to H4K12 hyperacetylation.

### Transcriptional characterization of mitochondrial dysfunction- and H4K1ac-induced gene expression alteration in MitoParks

**Figure 8:**
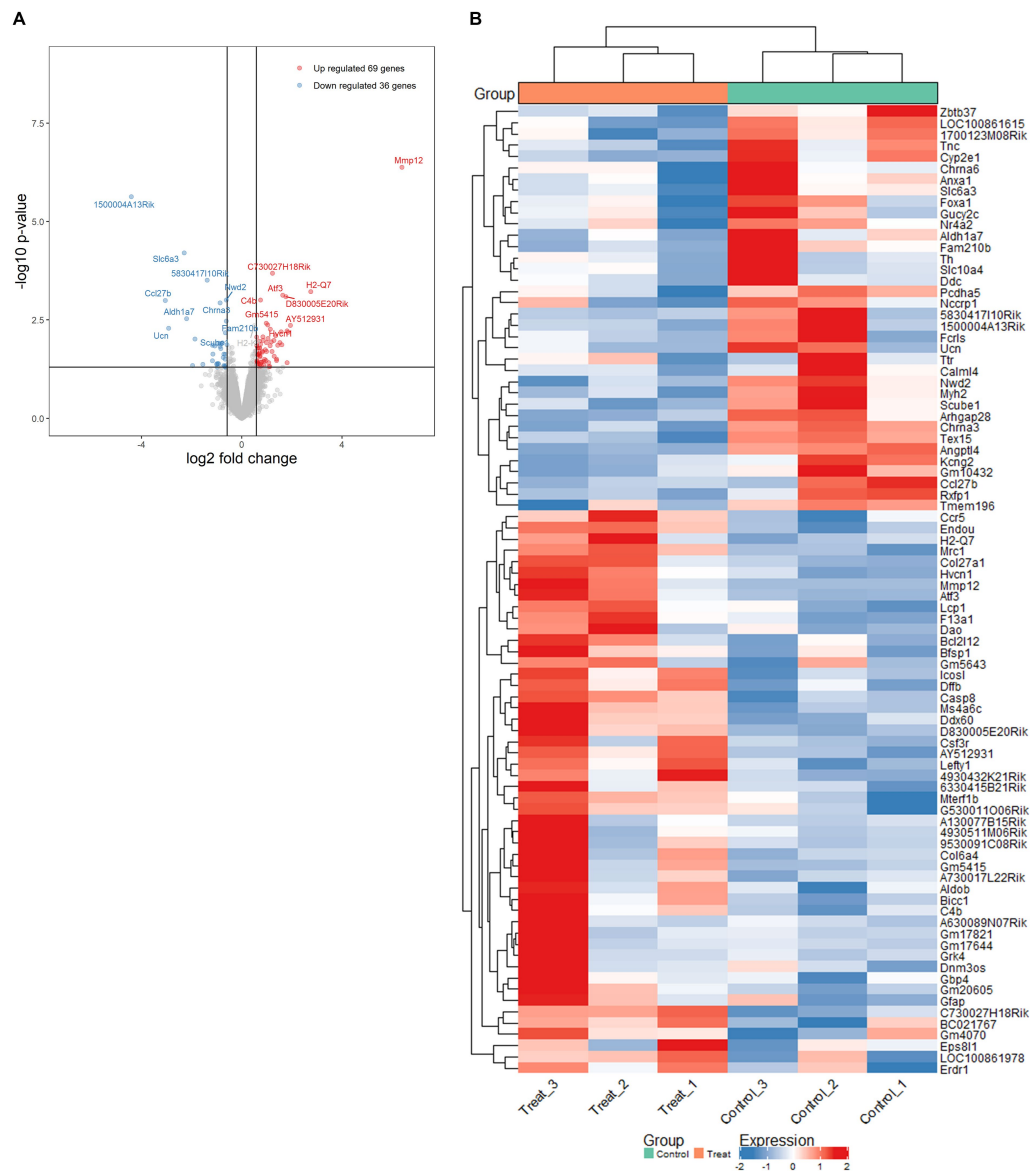
Mitochondria stress-induced transcriptomic alterations were profiled by RNA-seq in the SN of MitoPark mice. **(A)** Volcano plot of differentially expressed genes (DEGs) shows significance versus fold-change on the *y* and *x* axes, respectively. The top 15 significant genes were labelled. **(B)** Heat map shows the data of top DEGs where the individual values contained in a matrix are represented in colors. Red means high expression and blue means low expression.

**Figure 9:**
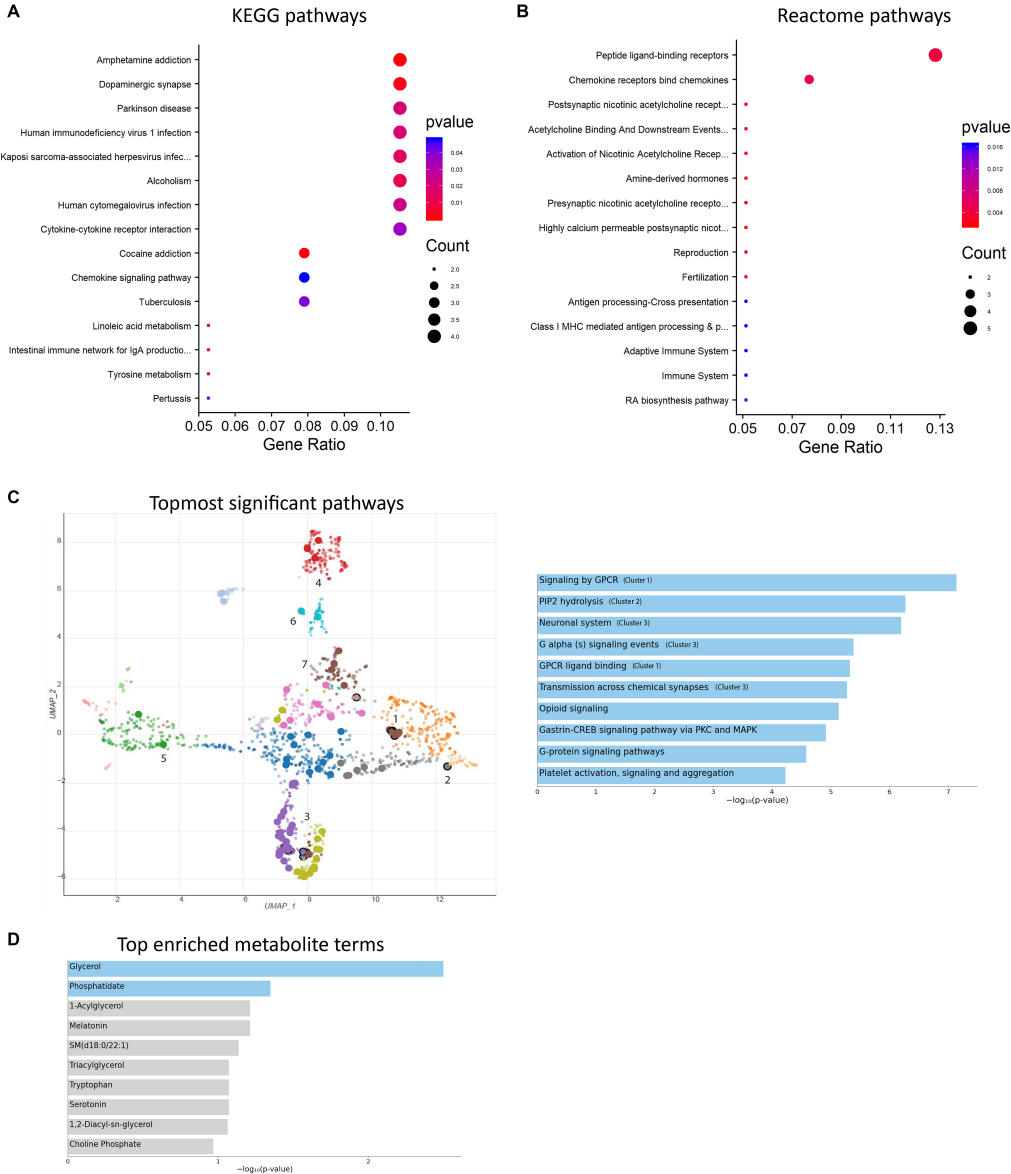
Enrichment analyses were performed. **(A)** KEGG pathway bubble plot shows the topmost 15 significant pathways associated with DEGs compared with the whole genome background. **(B)** Reactome pathway bar plot displays the topmost 15 gene-clustered pathways of a ranked list. **(C)** UMAP representation (left) and the topmost 10 significant pathways (right, labeled with clusters 1–3). Cluster 4: lipoprotein metabolism; cluster 5: mitochondrial gene expression; cluster 6: glycerolipid metabolism; cluster 7: dopamine receptor. **(D)** Top enriched metabolite terms. The top 10 enriched terms are displayed based on the -log10(*p*-value).

To further investigate the alterations in gene expression associated with mitochondrial dysfunction-mediated H4K12ac modification, which may underlie disease processes, we performed RNA-seq analyses on samples from the SN of 17-week MitoParks and littermate controls. Volcano plot analysis between three MitoParks and three littermate controls showed differentially expressed genes (DEGs) with significant change, including 69 upregulated genes and 36 downregulated genes (Figure 8A). Among top upregulated DEGs (Figure 8B), *Hvcn1* encodes the voltage-gated H^+^ channel and enables NADPH oxidase (NOX) function by compensating cellular loss of electrons with protons, which are required for phagocytosis, and thus HVCN1 was required for NOX-dependent ROS generation (Seredenina et al., 2015). *Mmp12* has been associated with PD risk in a Polish population (Białecka et al., 2017). *Atf3* was found to be transcriptionally deficient and associated with the antioxidant response pathway in hESC-derived *SNCA-*A53T human DAergic neurons and the SN from PD patients (Czaniecki et al., 2019). *H2-q7* is a major histocompatibility complex (MHC) I class member, while *C4b* is a major fragment for activation of the complement system. Intermediate filaments are the major cytoskeletal proteins in neurons, and *Bfsp1* co-assembles with Bfsp2 to form filaments (Muñoz-Lasso et al., 2020). *Dffb* codes caspase-dependent DNase, which triggers both DNA fragmentation and chromatin condensation during apoptosis. For gene expression patterns, a KEGG enrichment plot (Figure 9A) of the upregulated and downregulated DEGs in MitoParks highlight the pathways involved in dopamine process, neuroinflammation (e.g., cytokine-cytokine receptor interaction and chemokine signaling pathway), and the metabolic process. Reactome pathway enrichment analysis shows that peptide ligand-binding receptors were the most significant pathway with the largest number of DEGs (Figure 9B). With a focus on upregulated genes, Figure 9C shows the enriched clusters including GPCR signaling, metabolism process (e.g., lipid, lipidprotein, and glycerolipid), mitochondrial gene expression, dopamine receptor (DEGs of the top clusters are shown in Supplementary Figure S6). For metabolomic analysis, glycerol metabolite has the most significant overlap with the input query gene set, which agrees with our previous publication in MitoParks (Ghaisas et al., 2019) (Figure 9D). Collectively, our RNA-seq results demonstrate energy, mitochondria, and dopamine association with MitoPark in neurodegeneration.

## Discussion

Epigenetic marks have been implicated in the perturbation of DAergic cellular homeostasis (Song et al., 2011; Huang et al., 2021). They are thought to be sensitive to environmental stimuli and measurable across human populations (Berdasco and Esteller, 2019). Our current study profiled genome-wide acetylation sites for the core histone H4 and, to our best knowledge, we identified for the first time H4K12ac as a key epigenomic mark in environmentally-linked PD models. The high fold-change (352.3-fold) renders H4K12ac to be a remarkable site relative to the many other sites on H4. Our findings were extended to MitoPark mice and postmortem PD SN tissues and sections for adding etiological and clinical relevance to our hypothesis that mitochondrial stress-induced H4K12ac plays an important role in PD.

Though we do not fully understand the contribution of H4K12ac to PD etiology, it is important to note that H4K12ac is also implicated in other cell types and neurodegenerative conditions. For example, H4K12ac levels selectively decline with aging and have been associated with memory impairment in aged animals (Peleg et al., 2010). Increased H4K12ac has also been observed in monocytes of AD mice and human patients (Plagg et al., 2015), hippocampal neurons and in mouse forebrain primary neuronal cultures (Wee et al., 2014; Ghosh et al., 2016), and post-mortem HD brains (Narayan et al., 2020). Given the certain similarities between neurodegenerative diseases, these findings highlight the potential of H4K12ac to be the enrichment mark specific to the progression of neurodegenerative diseases like AD, HD and PD. Interestingly, the elevated H4K12ac deposition we documented in the SN of human PD brains is coincidentally supported by the results of a prefrontal cortex study of PD from the Netherlands Brain Bank (NBB) and the Norwegian ParkWest study (PW) (Toker et al., 2021). Again, this evidence demonstrates that H4K12ac has potential to be an epigenetic mark signature for PD pathology or progression. The sensitivity of H4K12ac to low-dose, chronic paraquat treatment further suggests its potential as an early indicator of environmentally linked PD. Considering that H4K12ac and the other highly modified deposition site H3K27ac occur at slightly different timepoints (Huang et al., 2021), using a composite of H4K12ac and H3K27ac marks with other auxiliary diagnosis methods could be a good option for pathological diagnosis or progression prediction.

While H4K12ac mainly affects transcriptional regulation, there is emerging evidence suggesting crosstalk or indirect interactions between histone modifications and mitochondrial proteins through metabolites, such as acetyl-CoA and α-ketoglutarate in the tricarboxylic acid (TCA) cycle (Santos, 2021; Shi et al., 2021). However, it was not until recently that the epigenomic outcomes of mitochondrial dysfunction were extensively studied, showing mitochondria’s far-reaching impact on epigenetics including histone acetylation (Shi et al., 2021; Li et al., 2022; Ohshima et al., 2022). Mitochondrial proteins primarily function in energy production, metabolism, and cellular signaling. Histone acetylation like H4K12ac plays a role in gene expression by modulating chromatin accessibility and transcription factor binding. Any potential direct interaction between H4K12ac and mitochondrial proteins would likely involve mechanisms, such as altered transcriptional programs or signaling cascades, that affect mitochondrial function through changes in gene expression. Research into the broader impacts of histone acetylation like H4K12ac on cellular metabolism and mitochondrial function may reveal potential cellular pathologies in PD.

Histone modifications change consistently in response to environmental stimuli. Despite their impact on gene expression, they bookmark the genome with prior experience (e.g., environmental insult counter) without altering the DNA sequence. With this in mind, for future studies, we are curious to what extent lifelong experience/exposure can be accumulated, how many marked nucleosomes and units of histone acetylation occur before the cumulative epigenomic impacts trigger PD onset. Unlike histone hypermethylation, histone hyperacetylation is relatively dynamic and active, increasing its therapeutic potential for PD treatment depending to what extent its cumulative effects can be undone.

Previous data shows reduced levels of HDAC4 upon exposure to the environmental toxicant paraquat (Song et al., 2011; Mielcarek et al., 2013). To investigate HAT/HDAC homeostasis in this study, we applied neurotoxicant treatments to DAergic N27 neuronal cells and identified HDAC4 along with HDAC2 as modulators for acetylation dysregulation. As a Class IIa HDAC, HDAC4 plays an important role in synaptic function and neuronal degeneration (Li et al., 2012; Mielcarek et al., 2015; Lang et al., 2019). In addition, Mielcarek et al. (2015) reported that HDAC4 possesses multiple cellular functions and is regulated by UPS. Similarly, we report here that proteasome dysfunction comes along with aberrant HDAC4. Also, according to Paroni et al. (2008), HDAC4 can be regulated by protein phosphatase 2A (PP2A) through dephosphorylation, whose N-terminus interacts with the catalytic subunit of PP2A. Unlike HDAC2, HDAC4 can shuttle between the nucleus and the cytoplasm, acting as a transcription repressor in gene expression. As reported here, our cell models did not show nuclear accumulation of HDAC4 as some publications have reported (Li et al., 2012; Lang et al., 2019), but we did observe this phenomenon in our other studies of cells treated with the neurotoxicants H_2_O_2_ and MPP^+^ (data not shown). This pattern is possibly attributed to the property of HDAC4, whose response depends on the toxicity and surrounding conditions. Hence, investigating other experimental PD models in the future would enable us to provide further information on epigenetic mark writers, erasers, and readers.

Regarding DEG functions in PD pathology, future studies should be conducted to identify which genes are specifically required for H4K12ac deposition and how those genes contribute to PD etiology, including conventional gene-editing methods, such as gain- and loss-of-function mutations. Enhanced CRISPR/Cas9 techniques (e.g., dCas9) can engineer epigenomic modification to perform promoter/enhancer silencing/activation, the manipulation of gene regulation, and mechanistic exploration of epigenetic inheritance (Hilton et al., 2015; Kearns et al., 2015; Zlotorynski, 2015; Nuñez et al., 2021). As spatial transcriptomic techniques come of age, empowering neuroscientists to capture RNA from histological tissues and visualize mRNA distribution in the targeted brain regions (Vickovic et al., 2019), the application of spatial transcriptomics to PD pathological studies would be beneficial to link PD-specific transcriptomic signatures to their spatial context in neural circuits. Moreover, since epigenetic writers, readers, and players have unique expression patterns in certain regions, the technique will help us to identify their respective enrichments in the SN and striatum, pushing the mechanistic study forward from two to three dimensional. This would enable us to look beyond cellular physiological function to reveal epigenetic modification in the context of circuit connectivity and their crosstalk.

Although no pharmaceuticals are available that can specifically regulate the levels of H4K12ac, several strategies could be considered: (1) developing small molecule HAT inhibitors and HDAC activators that specifically target the acetylation of H4K12 could be a promising approach. This would involve high-throughput screening of compound libraries to identify potential candidates. Please note that several HDAC inhibitors are already approved for clinical use in various cancers (e.g., vorinostat, romidepsin). These inhibitors could be repurposed or further developed to target neurodegenerative diseases by modulating H4K12ac levels. (2) Utilizing gene therapy techniques to modulate the expression of enzymes involved in H4K12 acetylation, such as the specific HAT and HDAC that regulate H4K12ac levels. (3) Employing CRISPR-dCas9 technology for precise targeting of H4K12ac at specific genomic loci, thereby modulating gene expression in a controlled manner.

Owing to the limitation of current technology, neuroscientists are still unable to track the dynamic alteration of histone acetylation during exposure to neurotoxicants in real time. To cease disease progression or to start treatments at presymptomatic stages and to identify at-risk individuals, we need indicators to diagnose the onset of PD (Emamzadeh and Surguchov, 2018). Recent clinical reports recognized potential epigenetic biomarkers in the blood of PD patients (Wang et al., 2019; Go et al., 2020; Nabais et al., 2021). To build upon our acetylation findings, our lab will also be investigating histone methylation to unravel the full story of epigenetic dysregulation in PD pathogenesis since through methylation or acetylation, genes switch on or off between activation or repression, and if a site is methylated, it cannot be acetylated.

Overall, our study further characterizes the “mitochondrial stress—H4K12ac axis” to comprehensively unravel a novel signaling pathway underlying the etiology of PD. It provides evidence for the neuroepigenetic mechanism of PD pathogenesis and identifies H4K12ac as a potential pharmacoepigenomic mark as a candidate for therapeutic translation.

## Data availability statement

The datasets presented in this study can be found in online repositories. The names of the repository/repositories and accession number(s) can be found below: https://www.ncbi.nlm.nih.gov/geo/, GSE180408.

## Ethics statement

Ethical approval was not required for the studies on humans in accordance with the local legislation and institutional requirements because the samples used were from a previous study where approval had been obtained. The animal study was approved by the Institutional Animal Care and Use Committee (IACUC) at ISU. The study was conducted in accordance with the local legislation and institutional requirements.

## Author contributions

MH: Conceptualization, Data curation, Formal analysis, Investigation, Methodology, Resources, Software, Validation, Visualization, Writing – original draft, Writing – review & editing. HJ: Writing – review & editing. VA: Supervision, Writing – review & editing. AK: Supervision, Writing – review & editing. AGK: Conceptualization, Funding acquisition, Project administration, Supervision, Writing – review & editing.
