# Supplementary material for: Mitochondrial stress-induced H4K12 hyperacetylation dysregulates transcription in Parkinson’s disease
Source: Front Cell Neurosci. 2024 Aug 12;18:1422362. doi: 10.3389/fncel.2024.1422362 (PMC11345260; doi:10.3389/fncel.2024.1422362)
Supplement: Supplementary file 1 [file Data_Sheet_1.PDF]

Supplementary Materials

Mitochondrial Stress-induced H4K12 Hyperacetylation Dysregulates Transcription in Parkinson's Disease

Authors

Minhong Huang[1#], Huajun Jin[2], Vellareddy Anantharam[2], Arthi Kanthasamy[2], Anumantha G. Kanthasamy[1,2*]

Affiliations

[1]Parkinson Disorders Research Laboratory, Iowa Center for Advanced Neurotoxicology, Department of Biomedical Sciences, 2062 Veterinary Medicine Building, Iowa State University, Ames, IA 50011

[2]Center for Neurological Disease Research, Department of Physiology and Pharmacology, 325 Riverbend Road, University of Georgia, Athens, GA 30602

# Present affiliation: Department of Molecular Pharmacology and Experimental Therapeutics, 200 1st St SW, Mayo Clinic, Rochester, MN 55905

***Correspondence:** Anumantha Kanthasamy, Professor, Johnny Isakson Chair, Georgia Research Alliance Eminent Scholar, and Director, Center for Neurological Disease Research, Department of Physiology and Pharmacology, 325 Riverbend Road, Center for Molecular Medicine Blgd, University of Georgia, Athens, GA 30602. Telephone: (706) 542-2380; Fax: (706) 542-4412; Email: anumantha.kanthasamy@uga.edu

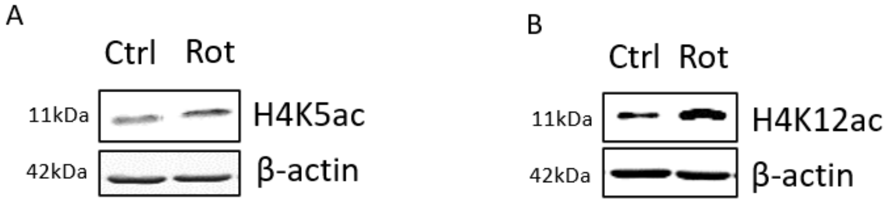

Supplementary Figure 1. **H4K12ac and H4K5ac deposition elevated in rotenone (Rot)-exposed N27 cells.** (A) Representative immunoblots for H4K5ac in Rot-treated N27s, and (B) representative immunoblots for H4K12ac with the same treatment.

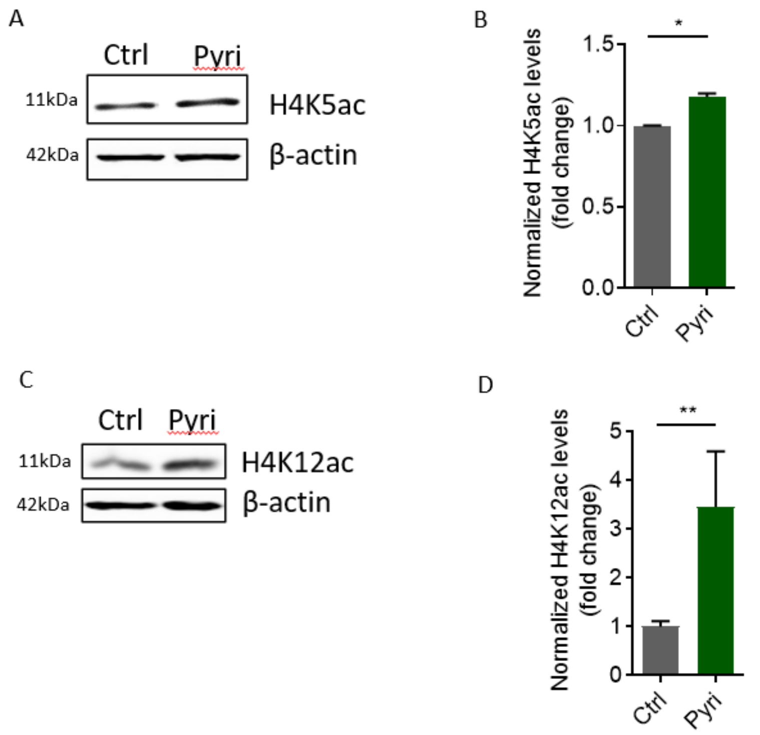

Supplementary Figure 2. H4K5ac and H4K12ac deposition elevated in pyridaben (Pyri)-exposed N27 cells. (A) Representative immunoblots for H4K5ac in Pyri-treated cells and (B) their quantification (n=2). (C) epresentative immunoblots for H4K12ac with the same treatment together with (D) their quantification (n=5-6). Error bars show mean ± s.e.m of unpaired two-tailed t tests. ns, not significant; *p<0.05; **p<0.01.

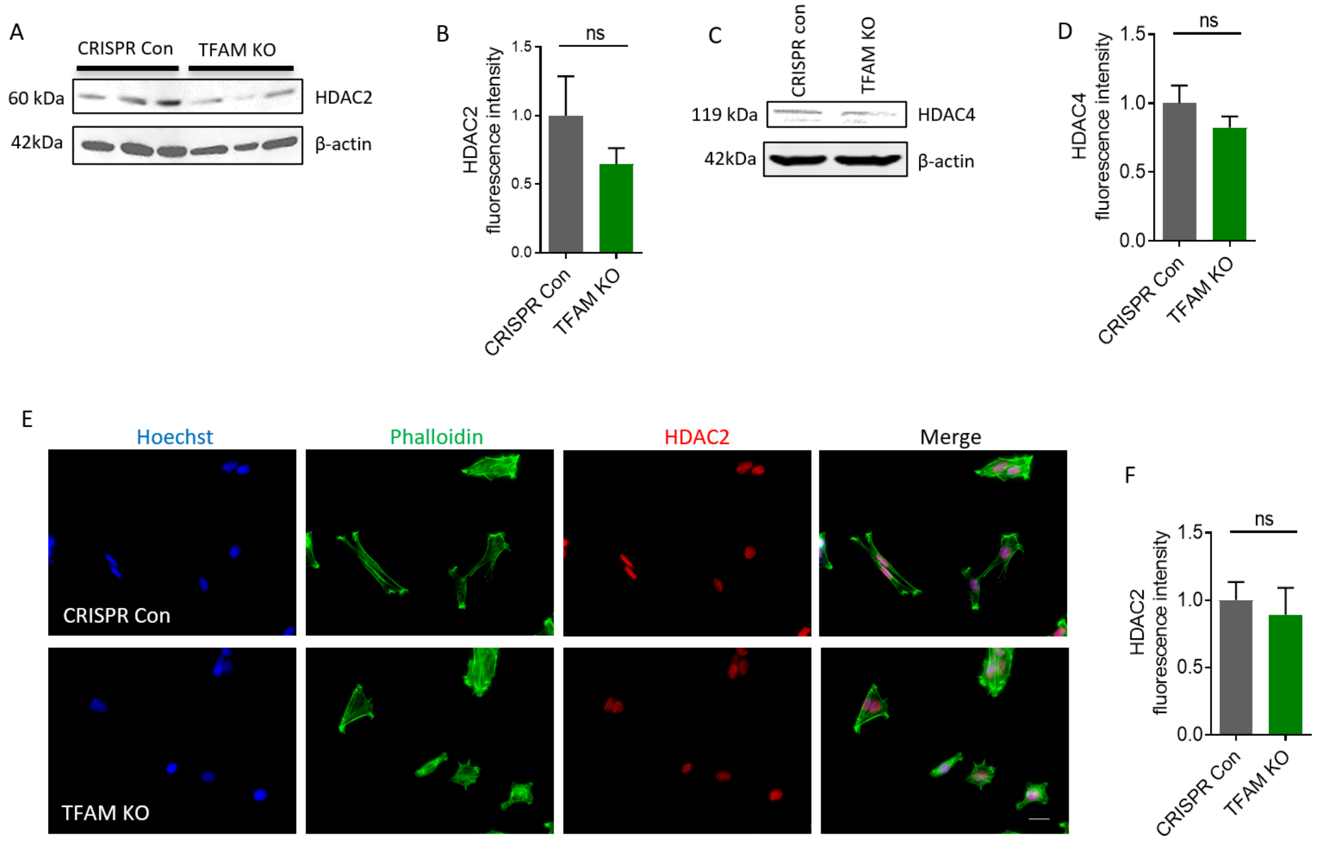

Supplementary Figure 3. **Translational levels of HDAC2 and HDAC4 decrease in TFAM-KO N27 cells.** (A) Immunoblots for HDAC2 in TFAM-KO N27 cells and (B) their quantification (n=3). (C) Representative immunoblots for HDAC4 in TFAM-KO N27 cells and (D) their quantification (n=8). Error bars show mean ± s.e.m of unpaired two-tailed t tests. ns, not significant.

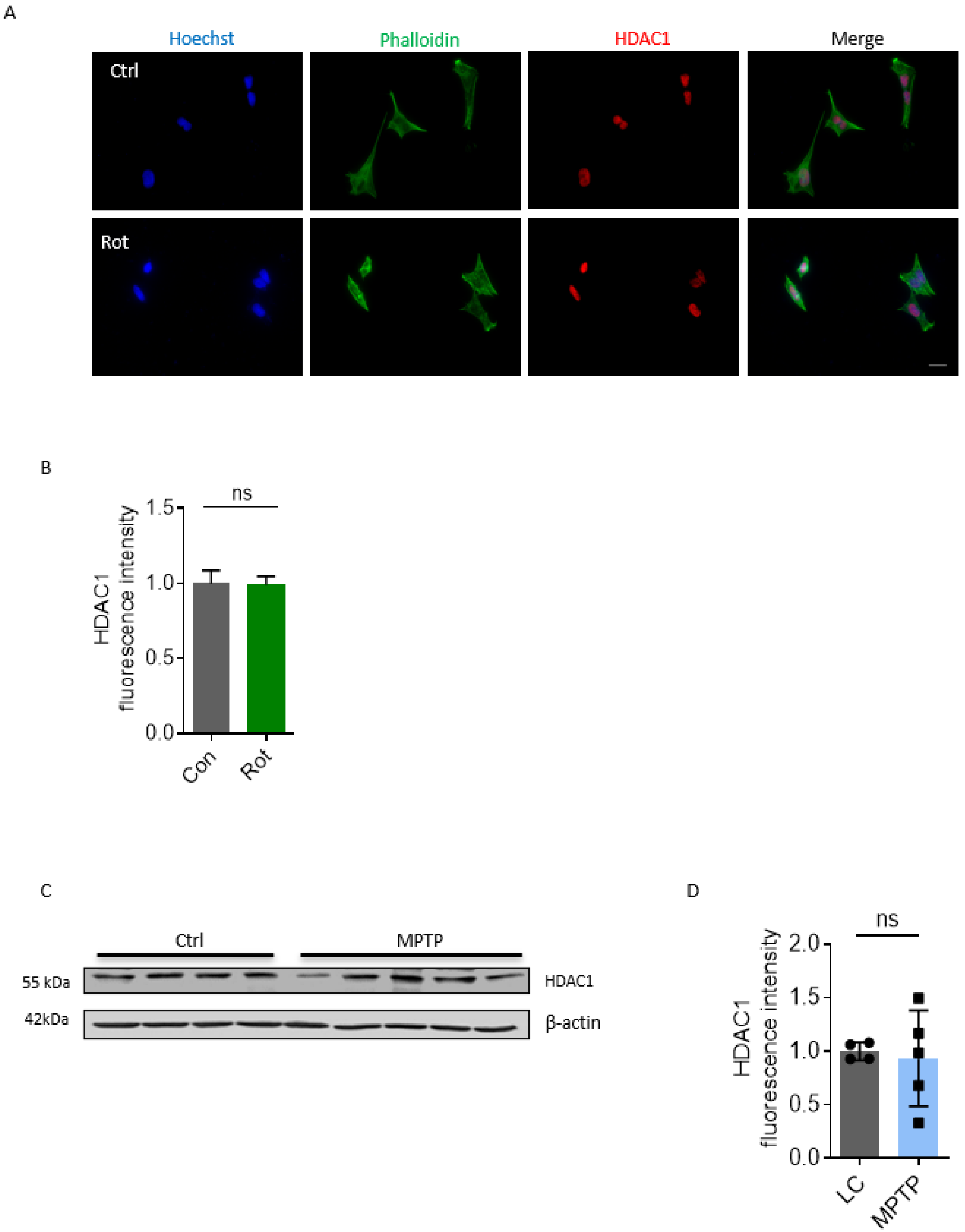

Supplementary Figure 4. HDAC1 showed no significant change in rotenone (Rot)-treated N27 cells and MPTP-treated C57BL/6 mice. (A) Immunohistochemistry for HDAC1 (red) and (B) its quantification in Rot-treated N27 cells (n=6). Independent experiments were repeated three times. (C) Immunoblots for HDAC1 in MPTP-treated C57BL/6 mice and (D) their quantification (n=4~5). Error bars show mean ± s.e.m of unpaired two-tailed t tests. ns, not significant.

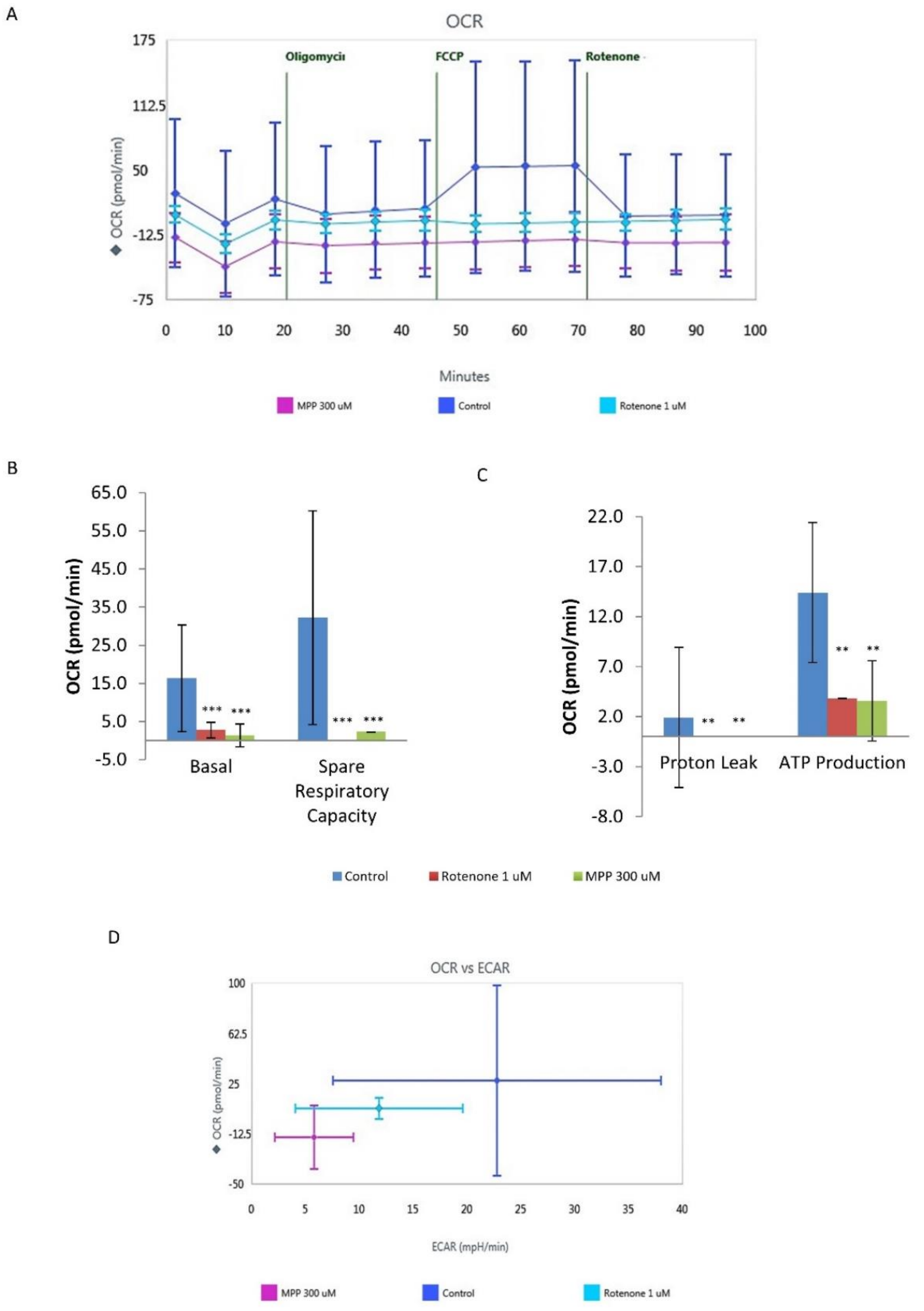

Supplementary Figure 5. **Impaired mitochondrial bioenergetics were analyzed in MPP-treated N27s.** (A, B, and C) Impaired mitochondrial bioenergetics via XFe24 Seahorse mitochondrial stress test displayed as (A) Respiration plot, (B) basal respiration and spare respiratory capacity, and (C) proton leak and ATP production (n=3). (D) Cellular phenotype plot comparing OCR on the y-axis and ECAR on the x-axis. Error bars represent mean ± s.e.m. of one-way ANOVA followed by Tukey's post hoc test. ns, not significant; **p<0.01; ***p<0.001.

term	p-value	q-value	overlap_genes
Signaling by GPCR	7.192183e-08	0.000038	[OPRD1, PDE1B, DGKB, ITPR1, RASGRP2, MCHR1, ADCY5, PPP3CA, GRM5, GNG7, PENK, DRD1, CGA, DRD2, TAC1, RGS9, WNT2, PRKCH, FZD2, ANXA1, PDE2A, WNT7A, TACR1, PDYN, ADRA2C, PDE10A, ADORA2A, CAMK4, PPP1R1B, AVP, PDE7B, NGEF, MGLL, DGKI]
PIP2 hydrolysis	5.336210e-07	0.000111	[PRKCH, DGKB, ITPR1, RASGRP2, DGKI, MGLL]
Neuronal system	6.281219e-07	0.000111	[KCNG1, KCNJ4, KCNH4, ACTN2, CHAT, CACNA2D3, KCNA5, KCNAB1, AKAP5, ADCY5, SLC5A7, GNG7, CAMK4, KCNJ2, CACNG4, SLC18A3]
G alpha (s) signaling events	4.061192e-06	0.000466	[PDE10A, ADORA2A, PDE1B, GNG7, PDE2A, DRD1, AVP, PDE7B, CGA, ADCY5]
GPCR ligand binding	4.697878e-06	0.000466	[OPRD1, FZD2, ANXA1, WNT7A, TACR1, PDYN, ADRA2C, MCHR1, GRM5, ADORA2A, GNG7, PENK, AVP, DRD1, CGA, DRD2, TAC1, WNT2]
Transmission across chemical synapses	5.295439e-06	0.000466	[KCNJ4, SLC5A7, ACTN2, GNG7, CAMK4, CHAT, CACNA2D3, AKAP5, CACNG4, KCNJ2, SLC18A3, ADCY5]
Opioid signaling	7.319964e-06	0.000552	[PPP3CA, PDE1B, GNG7, CAMK4, PPP1R1B, ITPR1, PDYN, ADCY5]
Gastrin-CREB signaling pathway via PKC and MAPK	1.204435e-05	0.000795	[PRKCH, ANXA1, DGKB, GNG7, ITPR1, TACR1, AVP, RASGRP2, TAC1, MCHR1, MGLL, DGKI]
G-protein signaling pathways	2.611389e-05	0.001532	[PPP3CA, PRKCH, PDE1B, GNG7, ITPR1, AKAP5, PDE7B, ADCY5]
Platelet activation, signaling and aggregation	5.896063e-05	0.003113	[PRKCH, ACTN2, DGKB, ACTN1, GNG7, ALB, ITPR1, RASGRP2, ADRA2C, MGLL, DGKI]

Supplementary Figure 6. **Upregulated DEGs in top 10 significant pathways.** Data analysis using BioPlaenet 2019 follow the published protocol (*1*, *2*).

Reference

1. H. Zhang *et al.*, 3D CRISPR screen in prostate cancer cells reveals PARP inhibitor sensitization through TBL1XR1-SMC3 interaction. *Front Oncol* **12**, 999302 (2022).
2. R. Huang *et al.*, The NCATS BioPlanet - An Integrated Platform for Exploring the Universe of Cellular Signaling Pathways for Toxicology, Systems Biology, and Chemical Genomics. *Front Pharmacol* **10**, 445 (2019).
